# Dual regulation of metabolic reprogramming and protein competitive modifications: the core role and mechanism of MRPL36 in glioblastoma malignant progression

**DOI:** 10.1186/s13046-026-03764-w

**Published:** 2026-06-18

**Authors:** Haonan Ye, Yuqi Wen, Zhihua Chen, Lei Wu, Zhe Zhang, Jie Zeng, Siyi Zhao, Bowen Fan, Bin Liao, Xinyu Tu, Zhangda Xiong, Lieliang Zhang, Youyi Xia, Jun Xie, Hong Zhu, Hua Guo, Feng Xiao

**Affiliations:** 1 Department of Neurosurgery, The Second Affiliated Hospital, Jiangxi Medical College, Nanchang University, 1# Minde Road, Nanchang, Jiangxi 330006 China; 2 Jiangxi Key Laboratory of Neurological Tumors and Cerebrovascular Diseases, Nanchang University, Nanchang, Jiangxi 330006 China; 3 JXHC key Laboratory of Neurological medicine, Nanchang University, Nanchang, Jiangxi 330006 China; 4 Institute of Neuroscience, Nanchang University, Nanchang, Jiangxi 330006 China; 5 Jiangxi Province Key Laboratory of Neurological Diseases, Nanchang University, Nanchang, Jiangxi 330006 China; 6 Department of Emergency Medicine, The Second Affiliated Hospital, Jiangxi Medical College, Nanchang University, Nanchang , China; 7 Department of Anesthesiology, The Second Affiliated Hospital, Jiangxi Medical College, Nanchang University, Nanchang, China; 8 Department of Gastrointestinal Surgery, The Second Affiliated Hospital, Jiangxi Medical College, Nanchang University, Nanchang, Jiangxi 330006 China; 9 Department of Ophthalmology, The Second Affiliated Hospital, Jiangxi Medical College, Nanchang University, Nanchang, Jiangxi 330006 China

**Keywords:** Ferroptosis, Palmitoylation, Ubiquitination, MRPL36, GPX4

## Abstract

**Background:**

Glioblastoma (GBM) has an extremely poor prognosis. Abnormal mitochondrial function, metabolic disorders, and dysregulated ferroptosis all drive its malignant progression, and the underlying molecular regulatory mechanisms still need further clarification. This study focuses on the regulatory mechanisms linking mitochondrial function, lipid metabolism, and ferroptosis to provide new directions for clinical treatment.

**Methods:**

Bioinformatics analysis confirmed aberrant upregulation of mitochondrial ribosomal protein L36 (MRPL36) in GBM, correlating with poor prognosis. In vitro and in vivo assays validated its roles in regulating GBM cell proliferation and invasion. RNA sequencing and untargeted metabolomics were used to analyze molecular changes upon MRPL36 knockdown. Acyl-biotin exchange (ABE) assays examined MRPL36 regulation of zinc finger DHHC-type palmitoyltransferase 12 (ZDHHC12)-mediated glutathione peroxidase 4 (GPX4) palmitoylation. Co-immunoprecipitation (Co-IP), Western blotting and mass spectrometry (MS) characterized tripartite motif-containing 33 (TRIM33)-dependent K48-linked ubiquitination and proteasomal degradation of GPX4 under MRPL36 modulation. The molecular network driving GBM progression was ultimately clarified.

**Results:**

MRPL36 is highly expressed in GBM and associated with poor patient prognosis. Silencing MRPL36 exacerbates mitochondrial damage and disturbs lipid metabolism homeostasis. It suppresses ZDHHC12-mediated GPX4 palmitoylation, promotes TRIM33-mediated GPX4 K48-linked ubiquitination and degradation, induces ferroptosis in GBM cells, and markedly inhibits tumor proliferation, invasion, and in vivo growth. Ferroptosis inhibitors partially reverse these inhibitory effects, confirming that MRPL36 regulates GBM progression by balancing GPX4 palmitoylation and ubiquitination.

**Conclusion:**

This study confirms that MRPL36, as a key regulatory factor in GBM progression, maintains mitochondrial function and lipid metabolism homeostasis, modulates the balance between GPX4 palmitoylation (mediated by ZDHHC12) and ubiquitination (mediated by TRIM33), inhibits ferroptosis, and promotes GBM malignant progression, providing new targets and theoretical basis for GBM precise treatment.

**Supplementary Information:**

The online version contains supplementary material available at https://doi.org/10.1186/s13046-026-03764-w.

## Introduction

Glioblastoma (GBM) is the most common and malignant primary intracranial tumor in the adult central nervous system, associated with an extremely poor clinical prognosis [1, 2]. Despite the standard comprehensive treatment strategy based on surgical resection combined with temozolomide chemotherapy and radiotherapy, the median survival of patients remains less than 15 months, with a 5-year survival rate below 10% [3, 4]. Multiple obstacles inherent to GBM, including chemo-radiotherapy resistance, high postoperative recurrence rate, blood-brain barrier, invasive growth, and a unique immunosuppressive tumor microenvironment, render various emerging therapies such as immunotherapy and tumor-treating fields unable to achieve long-term benefits [5, 6]. Therefore, in-depth dissection of the core molecular regulatory network underlying GBM malignant progression and identification of key driving targets are of great scientific significance and clinical value for developing more precise and effective intervention strategies to improve patient prognosis.

Mitochondrial ribosomal protein L family (MRPL) proteins are located in the mitochondrial matrix and represent core components of the large subunit of the mitochondrial ribosome [7, 8]. As key regulators of mitochondrial function, MRPL family members are widely involved in core biological processes including mitochondrial DNA transcription, mRNA translation, and ribosome assembly [9, 10]. They exert critical regulatory roles in mitochondrial gene expression, energy metabolic reprogramming, oxidative stress response, and tumorigenesis and progression [11–13]. As an important member of this family, mitochondrial ribosomal protein L36 (MRPL36) is primarily engaged in mitochondrial protein synthesis and is essential for maintaining mitochondrial structural integrity, oxidative phosphorylation efficiency, and cellular energy homeostasis [8, 14]. Aberrant expression or dysfunction of MRPL36 can trigger mitochondrial metabolic disorders and is closely associated with the development and progression of various diseases.

Ferroptosis is an iron-dependent form of programmed cell death characterized by massive accumulation of lipid peroxides [15, 16]. Accumulating evidence confirms that dysregulated ferroptosis plays a pivotal role in malignant tumor progression [17–19]. Iron overload, lipid peroxidation, and other stresses can inhibit the activity of glutathione peroxidase 4 (GPX4), resulting in impaired clearance of lipid peroxides and ultimately triggering ferroptosis [20, 21]. Tumor cells often acquire survival advantages and enhance therapeutic resistance by evading ferroptosis, thereby facilitating cancer progression [22, 23]. Post-translational modification of GPX4 represents a critical mechanism governing its protein stability and ferroptosis sensitivity, and contributes significantly to ferroptosis evasion and malignant progression in glioma [24, 25].

This study demonstrates that MRPL36 promotes the malignant progression of GBM by regulating ZDHHC12-mediated palmitoylation of GPX4 at cysteine 66 (C66). This modification antagonizes TRIM33-mediated K48-linked polyubiquitination and degradation of GPX4, thereby stabilizing GPX4 and suppressing ferroptosis. Using multi-omics analysis combined with functional experiments, we further verified that MRPL36 acts as a key driver in GBM progression and established the MRPL36-ZDHHC12/TRIM33-GPX4 axis as a critical signaling node linking mitochondrial function, protein post-translational modification, and the ferroptosis pathway. These findings provide novel mechanistic insights and potential strategies for precise targeted therapy of GBM.

## Materials and methods

### GBM data acquisition

In the present study, four independent GBM datasets were included, namely CGGA, GSE16011, GSE7696, and Rembrandt. Gene expression profiles and survival data of the CGGA dataset were obtained from the CGGA database (http://www.cgga.org.cn/), while data for the other three datasets were retrieved from the GEO database (https://www.ncbi.nlm.nih.gov/gds). Pan-cancer analysis and pan-cancer prognostic analysis were conducted using the SangerBox website (https://www.sangerbox.com/).

### Clinical samples collection

From 2020 to 2023, a total of 12 pairs of tumor tissues and matched para-cancerous tissues (PCTs) were resected from patients with GBM in the Department of Neurosurgery, the Second Affiliated Hospital of Nanchang University. After resection, the tissue samples were first fixed in 10% neutral buffered formalin, then dehydrated in 70% ethanol, and finally embedded in paraffin and sectioned. Informed consent was obtained from all enrolled GBM patients, and the study protocol was approved by the Ethics Committee of The Second Affiliated Hospital of Nanchang University (Review [2024] No. 21)

### IHC assay

Paraffin-embedded sections were first incubated with normal goat serum for 30 min to block non-specific binding. Primary antibodies were then applied, followed by incubation overnight at 4 °C. Biotin-labeled secondary antibodies were added and incubated for 60 min at room temperature. The avidin-biotin peroxidase system was used, and DAB chromogenic solution was added for visualization. Nuclei were counterstained with hematoxylin. Immunohistochemical staining was quantified using the H-score system ranging from 0 to 300. Staining intensity was divided into four grades: no staining, weak staining, moderate staining and strong staining, corresponding to scores of 0, 1, 2 and 3. The proportion of positive cells was recorded from 0% to 100%. Cells with staining intensity of 1, 2, or 3 were defined as positive. The final H-score was calculated as the sum of the products obtained by multiplying the staining intensity score by the percentage of positive cells at each corresponding intensity level. All tissue sections were independently evaluated by two experienced pathologists in a double-blinded manner, who were unaware of sample grouping and relevant clinical information.

### RNA extraction and qRT-PCR analysis

Total RNA was extracted from GBM cells using the Simply P Total RNA Extraction Kit (Bioflux, China). The isolated RNA was reverse-transcribed into cDNA using the MonScript RTIII All-in-One Mix with dsDNase (Monad, China). Real-time quantitative PCR (qPCR) was performed using the MonAmp RapidStart Universal SYBR Green qPCR Mix (Monad, China) to determine the mRNA expression levels of target genes. All primer sequences are listed below:

**Table:** 

Gene Name	Forward (5’-3’)	Reversed (5’-3’)
MRPL36 GPX4	AAGCGCTGCAAGGACTGTTA GAAGATCCAACCCAAGGGCA	CTTGTGCCTCGGATGGGTTT GACGGTGTCCAAACTTGGTG

### Cell culture

The GBM cell lines used in this study included U87MG, T98G, LN229, U118MG, and U251MG, all of which were purchased from the American Type Culture Collection (ATCC). These GBM cell lines were cultured in DMEM (Dulbecco’s Modified Eagle’s Medium, Gibco, USA) supplemented with 10% fetal bovine serum (FBS, Gibco, USA) and antibiotic-antimycotic mixture (Gibco, USA). The cells were maintained in a humidified incubator at 37 °C with 5% CO₂.

### siRNA transfection in GBM cells

When GBM cells reached 65%-70% confluence, target gene-specific siRNAs or negative control siRNAs were transfected into cells using Lipofectamine 3000 (Invitrogen, USA) according to the manufacturer’s protocol. Forty-eight hours post-transfection, the knockdown efficiency was confirmed by quantitative real-time PCR (qRT-PCR) and Western blotting.

### CCK-8 assay

Transfected GBM cells were seeded into 96-well plates at a density of 2 × 10^3^ cells per well and incubated overnight. Subsequent procedures were performed according to the manufacturer’s instructions for the CCK-8 kit (Gibco, USA). The optical density (OD) at 450 nm was measured daily using a microplate reader (Thermo Fisher, USA) for 4 consecutive days.

### Colony formation assay

Transfected GBM cells were seeded into 6-well plates at a density of 1000 cells per well. After 2 weeks of culture, the cells were fixed with 4% paraformaldehyde and stained with 0.1% crystal violet for observation.

### EdU assay

Transfected GBM cells were seeded into 24-well plates at a density of 2 × 10^4^ cells per well and incubated. EdU reagent (Beyotime, C0078S) was added and the cells were incubated for 2 h. The cells were fixed with 4% paraformaldehyde, permeabilized with 0.3% Triton X-100, and subjected to Click reaction and staining according to the manufacturer’s protocol. Nuclei were stained with Hoechst, followed by observation under a fluorescence microscope and calculation of the positive cell rate.

### Wound healing assay

Transfected GBM cells were seeded into 6-well plates. When cell confluence reached 90%, a wound scratch was created in the cell monolayer using a 200 µl sterile pipette tip. Wound images were captured at 0 h and 24 h to analyze cell migration ability.

### Transwell assay

For the migration assay, 6 × 10^4^ transfected cells resuspended in serum-free medium were seeded into the upper chambers of uncoated Transwell inserts. For the invasion assay, the upper chamber membranes were pre-coated with diluted Matrigel (Yeasen, China), and the same number of cells was seeded. Then, 500 µl of medium supplemented with 25% fetal bovine serum (FBS) was added to the lower chambers. After incubation at 37 °C for 24–48 h, non-migrated cells on the upper surface were removed. Following fixation and staining, migrated cells were observed and counted under a microscope to evaluate cell migration and invasion abilities.

### ROS and ferrous ion level assay

Transfected GBM cells were seeded into 24-well plates and incubated under routine culture conditions. Intracellular reactive oxygen species (ROS) levels were detected using a ROS Fluorometric Assay Kit (Invitrogen, EEA019), with all procedures performed strictly according to the manufacturer’s instructions: after incubation of cells with the fluorescent probe at 37 °C in the dark, intracellular ROS levels were observed and evaluated under a fluorescence microscope. For ferrous ion (Fe^2+^) level detection, FerroOrange Ferrous Ion Fluorescent Probe (Maokang Biotech, Cat. No. MX4559-24 µg) was used. Cells were stained following the manufacturer’s protocol, and the fluorescence intensity of intracellular Fe^2+^ was observed under a fluorescence microscope.

### MDA content assay

After collecting cells from each group, the cells were washed with pre-cooled PBS and lysed thoroughly with an appropriate volume of lysis buffer. The supernatant was obtained by centrifugation at 4 °C. The malondialdehyde (MDA) detection working solution was prepared strictly according to the manufacturer’s instructions of the Lipid Peroxidation Assay Kit (Beyotime, Cat. No. S0131S). The supernatant was mixed with the working solution and incubated in a boiling water bath for 15 min. After cooling to room temperature and centrifugation, the absorbance of the supernatant at 532 nm was measured. The MDA concentration in each sample was calculated based on the standard curve provided with the kit and normalized to the total protein content.

### GSH/GSSG content assay

After collecting cells from each group and washing with pre-cooled PBS, the cells were fully lysed with an appropriate volume of deproteinization solution. After incubation on ice and centrifugation, the supernatant was collected. The working solution was prepared according to the manufacturer’s instructions of the glutathione (GSH) and oxidized glutathione (GSSG) Assay Kit (Beyotime, Cat. No. S0053). The supernatant was mixed with the working solution and incubated at room temperature, and the absorbance at 412 nm was measured. The contents of GSH and GSSG in each sample were calculated based on the standard curve provided with the kit and normalized to the total protein concentration.

### JC-1 and MitoSOX fluorescent probe staining

GBM cells were washed three times with pre-cooled PBS to remove residual medium and then labeled with different fluorescent probes. For mitochondrial membrane potential detection, cells were incubated with JC-1 probe (5 µM; Thermo Fisher) in the dark at 37 °C for 25 min, while for mitochondrial superoxide detection, cells were incubated with MitoSOX Red probe (5 µM; Thermo Fisher) in the dark for 15 min. After MitoSOX Red staining, cells were fixed with 4% paraformaldehyde at room temperature for 10 min and then counterstained with DAPI (1:1000; Thermo Fisher) in the dark for 10 min to label the nuclei. To ensure accurate detection of mitochondrial membrane potential, JC-1-stained cells were directly subjected to live-cell imaging in fresh serum-free medium without fixation. All cell samples were mounted with anti-fade mounting medium (Beyotime, Cat. No. P0126-5 ml), and fluorescent images were captured using a Zeiss LSM 980 laser confocal microscope with ZEN 3.0 software.

### Flow cytometry

GBM cells were washed 3 times with pre-cooled PBS, then incubated with 5 µM JC-1 probe (Thermo Fisher) in the dark for 25 min and 5 µM MitoSOX Red probe (Thermo Fisher) in the dark for 15 min, respectively. After incubation, the cells were centrifuged, the supernatant was discarded, and the cells were resuspended in pre-cooled PBS. A flow cytometer was used with FITC and PE channels, and the experimental and control groups were detected simultaneously. For the JC-1 group, mitochondrial membrane potential changes were reflected by the green fluorescence intensity in the FITC channel; for the MitoSOX Red group, mitochondrial superoxide levels were represented by the mean red fluorescence intensity in the PE channel.

### RNA-Seq analysis

Total RNA was extracted from cells in each group using TRIeasy™ Total RNA Extraction Reagent (Yeasen Biotechnology, Cat. No. 10606ES60). Qualified total RNA samples were sent to Tsingke Biotechnology for high-throughput RNA sequencing (RNA-seq). Library construction and sequencing were performed on the Illumina platform according to the standard protocols.

### Untargeted metabolomics analysis

After nude mice were sacrificed by cervical dislocation, orthotopic tumor tissues from the mouse brain were immediately collected, frozen in liquid nitrogen, and stored at -80 °C until use. Metabolites were extracted using a methanol-chloroform-water mixture (75:25, v/v) with steel beads. Metabolomic analysis was performed using a Thermo Fisher Vanquish UHPLC system coupled with an Orbitrap Exploris 120 mass spectrometer. Chromatographic separation was carried out at 40 °C using an ACQUITY UPLC HSS T3 column (2.1 × 100 mm, 1.8 μm). Data were acquired in both positive and negative electrospray ionization (ESI) modes. Full MS scan resolution was set at 60,000 (m/z 100–1000), and tandem mass spectra were obtained using high-energy collisional dissociation (HCD) with a collision energy of 30%. Based on the above platform and parameters, differential metabolites in samples were analyzed.

### Transmission electron microscopy (TEM) analysis

GBM cells were fixed with 2.5% glutaraldehyde, and then prepared using standard protocols for transmission electron microscopy. Mitochondrial morphology and structure in GBM cells were observed under a transmission electron microscope.

### Western blotting analysis

Protein expression levels were determined by Western blotting analysis. Total protein was extracted from cells using radioimmunoprecipitation assay (RIPA) lysis buffer (Solarbio, China) supplemented with protease and phosphatase inhibitors, and protein concentration was quantified using the BCA method. Protein samples were separated by 10% SDS-PAGE and then transferred onto a PVDF membrane. The membrane was blocked with 5% non-fat milk at room temperature for 1 h, and then incubated with primary antibodies including MRPL36 (Invitrogen, #PA5-100804, 1:1000), GPX4 (CST, #52455, 1:1000), Acyl-CoA Synthetase Long-Chain Family Member 4 (ACSL4) (Proteintech, #22401-1-AP, 1:5000), UB (CST, #3936, 1:1000), 4-Hydroxynonenal (4-HNE) (Thermo Fisher, #A700-303-T, 1:1000), Flag (CST, #2368, 1:1000), and Myc (CST, #13987, 1:1000) overnight at 4 °C, followed by incubation with HRP-conjugated secondary antibodies at room temperature for 1 h. Protein bands were visualized using a chemiluminescence imaging system (Tanon, China), and quantitative analysis was performed using ImageJ software.

### Plasmid transfection

Cells were seeded into 6-well plates and cultured until they reached 75%-80% confluence. Flag-, His-, and Myc-tagged plasmids were transfected into cells using Lipofectamine 3000 (Invitrogen, USA). At 48 h post-transfection, the expression efficiency of the plasmids was verified by quantitative real-time PCR (qRT-PCR) and Western blotting analysis.

### Acyl-biotin exchange assay

Acyl-biotin exchange (ABE) assay was performed according to previously reported protocols [26]. Cells were divided into control and treatment groups. Control cells received an equal volume of DMSO for 2-Bromopalmitate (2-BP) controls or BSA solution for palmitic acid (PA) controls. Cells in treatment groups received independent treatments: 50 µM PA (HY-N0830, MCE) for 6 h or 60 µM 2-BP (HY-111770, MCE) for 6 h at 37 °C. After lysis and total protein extraction, samples were mixed with 20 mM NEM (Sigma, E3876) and shaken at 4 °C for 2 h to block free thiols. Next, specific antibodies against target proteins and magnetic beads were added to perform immunoprecipitation and purification. Then, 1 M hydroxylamine (HAM, Sigma, Cat. No. 431362) was added to the purified protein complexes and incubated at room temperature for 1 h, followed by addition of 5 µM biotin-BMCC and incubation at 4 °C for 1 h to accomplish biotinylation. The immunoprecipitation beads were washed three times with PBS containing 0.2% SDS to remove non-specific binding, and then resuspended in SDS loading buffer, followed by denaturation at 95 °C for 10 min. The denatured protein samples were separated by sodium dodecyl sulfate-polyacrylamide gel electrophoresis (SDS-PAGE) and subjected to Western blotting analysis.

### Co-immunoprecipitation (Co-IP) analysis

Co-IP was performed as follows. Cells were harvested and lysed for total protein extraction. Equal amounts of cell lysates were incubated with primary antibodies specific for target proteins with rotation at 4 °C overnight to ensure sufficient binding. The next day, pre-washed magnetic beads (Thermo Fisher, Cat. No. 88803) were added, followed by rotation at 4 °C for 6 h to capture immune complexes. The beads were then washed with PBS three times on a magnetic stand to remove non-specific binding proteins. After discarding the wash buffer, the beads were resuspended in SDS loading buffer and denatured at 95 °C for 10 min to elute proteins. The beads were separated using a magnetic stand, and the supernatant was collected for subsequent Western blotting analysis.

### Mass spectrometry analysis

To screen proteins interacting with GPX4, an integrated strategy combining co-immunoprecipitation with liquid chromatography-tandem mass spectrometry (Co-IP-LC-MS/MS) was used in this study. Briefly, total protein was extracted from glioblastoma cells and incubated with GPX4-specific antibody with rotation at 4 °C overnight. The next day, protein A/G magnetic beads were added to capture immune complexes, followed by rotation at 4 °C for 6 h. After extensive washes with washing buffer, the bound proteins were eluted from the beads. The eluted proteins were then reduced, alkylated, and digested with trypsin, followed by nano-liquid chromatography separation and mass spectrometry analysis of the peptide mixture. Raw mass spectrometry data were processed with corresponding software and searched against the UniProt database for protein identification. Candidate interacting proteins of GPX4 were screened based on fold enrichment and statistical differences.

### Establishment of intracranial xenograft mouse model

All animal procedures in this study were approved by the Animal Care and Use Committee of Nanchang University (Approval No. NCULAE-20221031035). Six-week-old male BALB/c nude mice (Crisbio, Shanghai, China) were used to establish intracranial xenograft models of GBM. Lentivirus-mediated MRPL36 knockdown U251MG cells and negative control cells were harvested and resuspended in pre-chilled PBS to prepare single-cell suspensions. Mice were anesthetized by isoflurane inhalation, and the injection site was set as 2 mm right of the bregma, 1 mm posterior, at a depth of 3 mm according to standard stereotaxic coordinates. A total of 5 µL of cell suspension containing 3 × 10^5^ luciferase-labeled cells was slowly injected into the right frontal lobe at a rate of 1 µL/min. After implantation, tumor growth in the mouse brain was dynamically monitored by measuring luciferase activity using an IVIS Lumina Series III imaging system (PerkinElmer, USA). In the GBM targeted therapy experiment, mice were randomly assigned to two groups and administered PBS or the ferroptosis inhibitor Ferrostatin-1 (Fer-1) *via* tail vein injection. Fer-1 was given at 8 mg/kg every three days for 21 consecutive days. This inhibitor was chosen for in vivo intervention due to its good blood-brain barrier permeability, which renders it suitable for pharmacological intervention in intracranial GBM models. The endpoint was defined when mice showed moribund signs including rapid body weight loss and motor dysfunction, and humane euthanasia was performed by cervical dislocation. After the experiment, mouse brains were quickly dissected and fixed in 4% paraformaldehyde for subsequent hematoxylin-eosin (HE) staining and IHC analysis.

### Statistical analysis

Comparisons between groups were performed using Student’s t-test, one-way analysis of variance (one-way ANOVA), or two-way analysis of variance (two-way ANOVA). Survival analysis was conducted using the two-tailed log-rank test. Statistical significance is indicated by *P < 0.05, **P < 0.01, and ***P < 0.001, with *P* < 0.05 considered statistically significant.

## Results

### Upregulation of MRPL36 is associated with poor prognosis in GBM

**Fig. 1:**
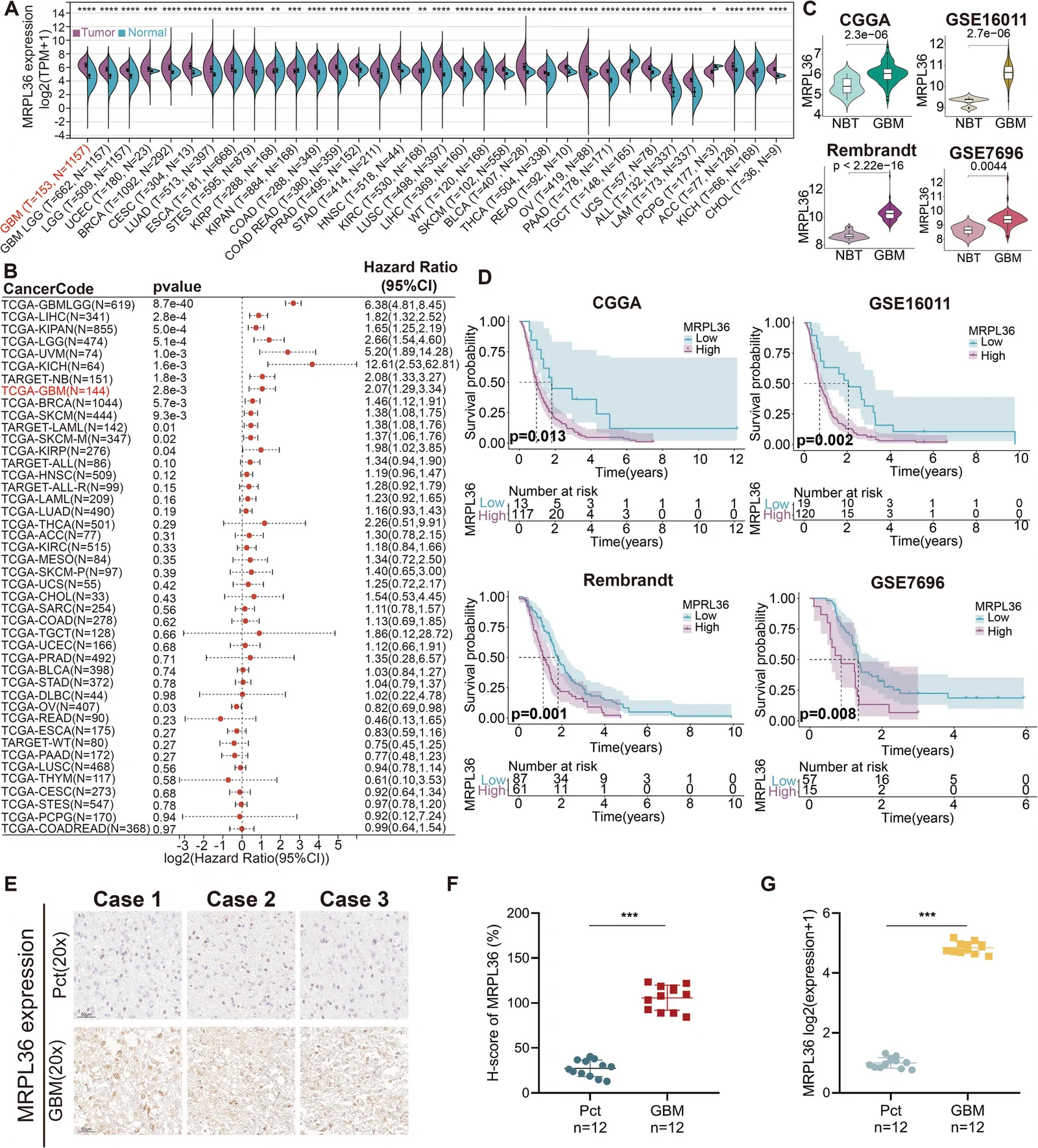
Overexpression of MRPL36 is associated with poor prognosis in GBM **A** Differential expression analysis of MRPL36 in various tumor tissues and their corresponding normal tissues. **B** Hazard ratio and 95% confidence intervals of MRPL36 expression for overall survival across multiple cancer types. (**C**) Differential expression analysis of MRPL36 in the CGGA, GSE16011, Rembrandt, and GSE7696 databases. (D) Prognostic analysis of MRPL36 expression stratified into low- and high- expression groups based on the CGGA, GSE16011, Rembrandt, and GSE7696 databases. The cutoff value represents the median expression level of MRPL36. **E**-**F** Immunohistochemical staining was used to evaluate the expression of MRPL36 in GBM tissues and paired peritumoral control tissues (PCTs) (**E**), and comparaison of H-scores between GBM tissues and PCTs (**F**). **G** qRT-PCR analysis of MRPL36 expression levels in GBM tissues and corresponding PCTs. *P < 0.05, **P < 0.01, ***P < 0.001

First, we performed pan-cancer expression analysis of MRPL36. The results showed that MRPL36 expression was significantly different between multiple cancer tissues and corresponding normal tissues. Notably, MRPL36 expression was significantly upregulated in GBM compared with normal tissues (Fig. 1A). Survival analysis indicated that high MRPL36 expression was generally associated with poor overall survival in various malignant tumors, and this correlation was particularly significant in GBM, suggesting that it markedly increased the risk of unfavorable patient prognosis (Fig. 1B).

Subsequently, we further validated the expression profile of MRPL36 using four independent GBM cohort datasets (CGGA, GSE16011, GSE7696, and Rembrandt). The results demonstrated that MRPL36 expression levels were significantly higher in GBM tissues than in normal brain tissues (NBT) across all four datasets (Fig. 1C). Survival analysis also revealed that GBM patients with high MRPL36 expression exhibited significantly worse prognosis than those with low expression in the above datasets (Fig. 1D).

In addition, immunohistochemical analysis was performed on 12 pairs of GBM tissues and paired PCTs, confirming that MRPL36 expression was significantly higher in GBM tissues than in PCTs (Figs. 1E-F). qRT-PCR results further verified that MRPL36 expression was significantly elevated in GBM tissues compared with PCTs (Fig. 1G). Thus, MRPL36 is highly expressed in GBM and associated with poor prognosis, suggesting a potential oncogenic role in GBM progression.

### MRPL36 promotes the malignant progression of GBM in vitro and in vivo

**Fig. 2:**
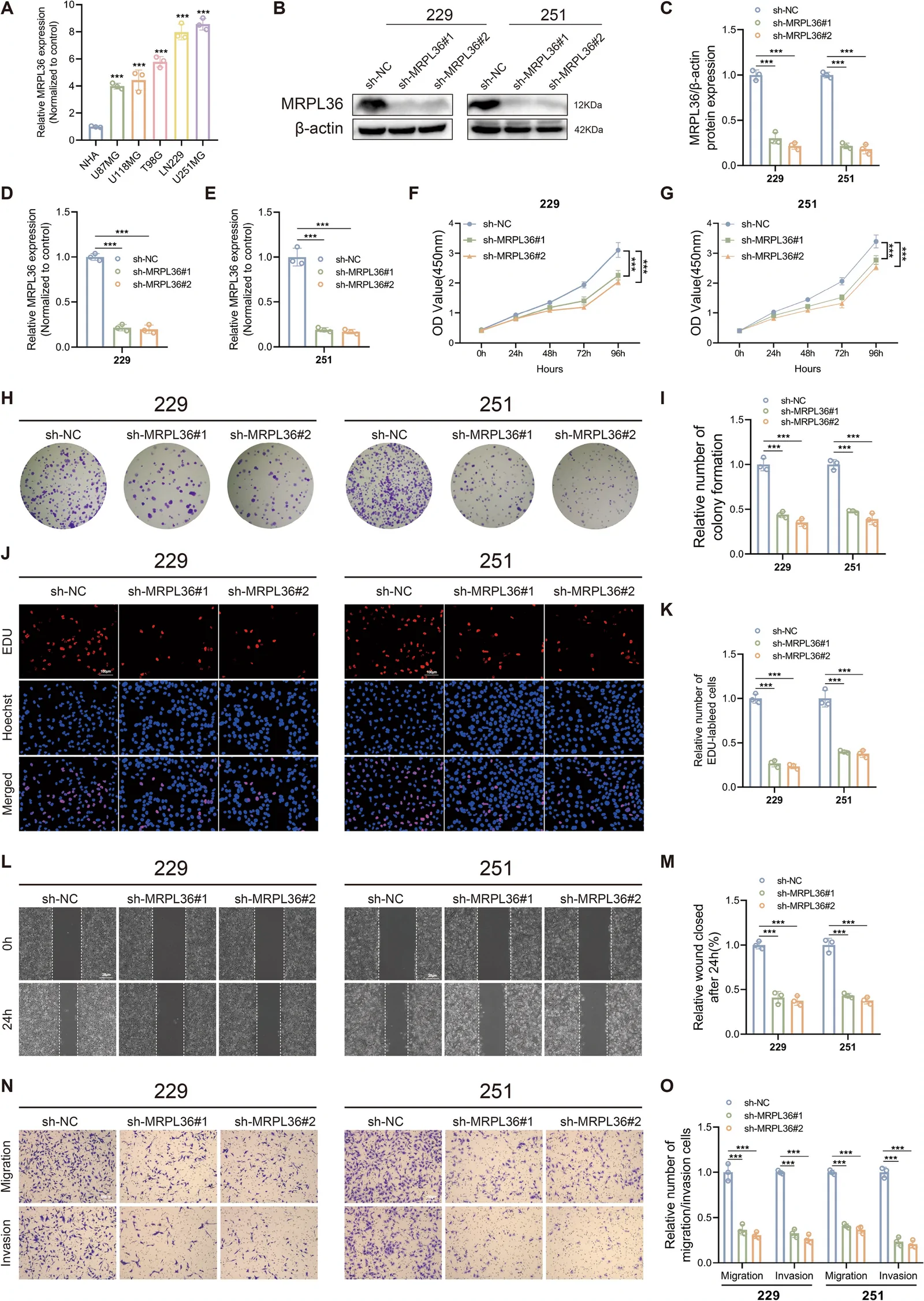
Knockdown of MRPL36 suppresses proliferation, migration, and invasion of GBM cells (**A**) Expression levels of MRPL36 in GBM cells and normal human astrocytes (NHA) analyzed by qRT-PCR. (**B**-**E**) Validation of MRPL36 knockdown efficiency in LN229 and U251MG cells by Western blotting (**B**, **C**) and qRT-PCR (**D**, **E**). (**F**-**G**) Cell viability of LN229 (**F**) and U251MG (**G**) cells after MRPL36 knockdown detected by CCK-8 assay. (**H**-**I**) Effect of MRPL36 knockdown on colony formation ability of LN229 and U251MG cells (**H**) and quantitative analysis of colony (**I**). (**J**-**K**) Representative images (**J**) and quantitative analysis (**K**) of EdU assays showing the proliferation of LN229 and U251MG cells after MRPL36 knockdown. (**L**-**M**) Representative images (**L**) and quantitative analysis (**M**) of wound-healing assays showing the migration ability of LN229 and U251MG cells after MRPL36 knockdown. (**N**-**O**) Representative images (**N**) and quantitative analysis (**O**) of Transwell assays showing the migration and invasion abilities of LN229 and U251MG cells after MRPL36 knockdown. *P < 0.05, **P < 0.01, ***P < 0.001

First, we examined the expression of MRPL36 in five GBM cell lines (U87MG, T98G, LN229, U118MG, and U251MG) and one normal human astrocyte cell line (NHA). The results showed that MRPL36 expression was significantly higher in GBM cell lines than in NHA cells, with the highest expression levels observed in U251MG and LN229 cells (Fig. 2A). Therefore, U251MG and LN229 cells were selected for subsequent experiments. To clarify the potential role of MRPL36 in the malignant progression of GBM, we performed a series of functional validation assays. In LN229 and U251MG cells, MRPL36 expression was stably downregulated using lentivirus-mediated specific shRNA, and MRPL36 stable knockdown cell lines were successfully established (Figs. 2B-E). CCK-8 assays revealed that MRPL36 knockdown markedly reduced the viability of LN229 and U251MG cells (Figs. 2F-G). Colony formation and EdU assays further confirmed that downregulation of MRPL36 significantly suppressed the clonogenicity and proliferation of GBM cells (Figs. 2H-K). These results indicate that MRPL36 promotes the proliferation of GBM cells in vitro. In addition, wound-healing and Transwell assays demonstrated that silencing MRPL36 significantly inhibited the migration and invasion of LN229 and U251MG cells (Figs. 2L-O). Collectively, MRPL36 promotes the proliferation, migration, and invasion of GBM cells in vitro.

To investigate whether MRPL36 knockdown affects the tumorigenesis of GBM in vivo, we established an in vivo model using immunodeficient nude mice (Supplementary Fig. 1A). The results showed that, compared with the sh-NC group, the sh-MRPL36#1 and sh-MRPL36#2 groups exhibited significantly smaller tumor volumes (Supplementary Fig. 1B-C), weaker overall fluorescence intensity (Supplementary Fig. 1D), a slower rate of body weight loss (Supplementary Fig. 1E), and longer overall survival time (Supplementary Fig. 1F). Furthermore, immunohistochemical analysis was performed on tumors from nude mice. The results showed that the proportion of Ki-67-positive cells was significantly decreased in tumor samples from the sh-MRPL36#1 and sh-MRPL36#2 groups compared with the sh-NC group (Supplementary Fig. 1G-H). In conclusion, knockdown of MRPL36 suppresses the growth of GBM in vivo.

### MRPL36 mediates mitochondrial-lipid metabolic crosstalk and ferroptosis to drive GBM malignant progression

**Fig. 3:**
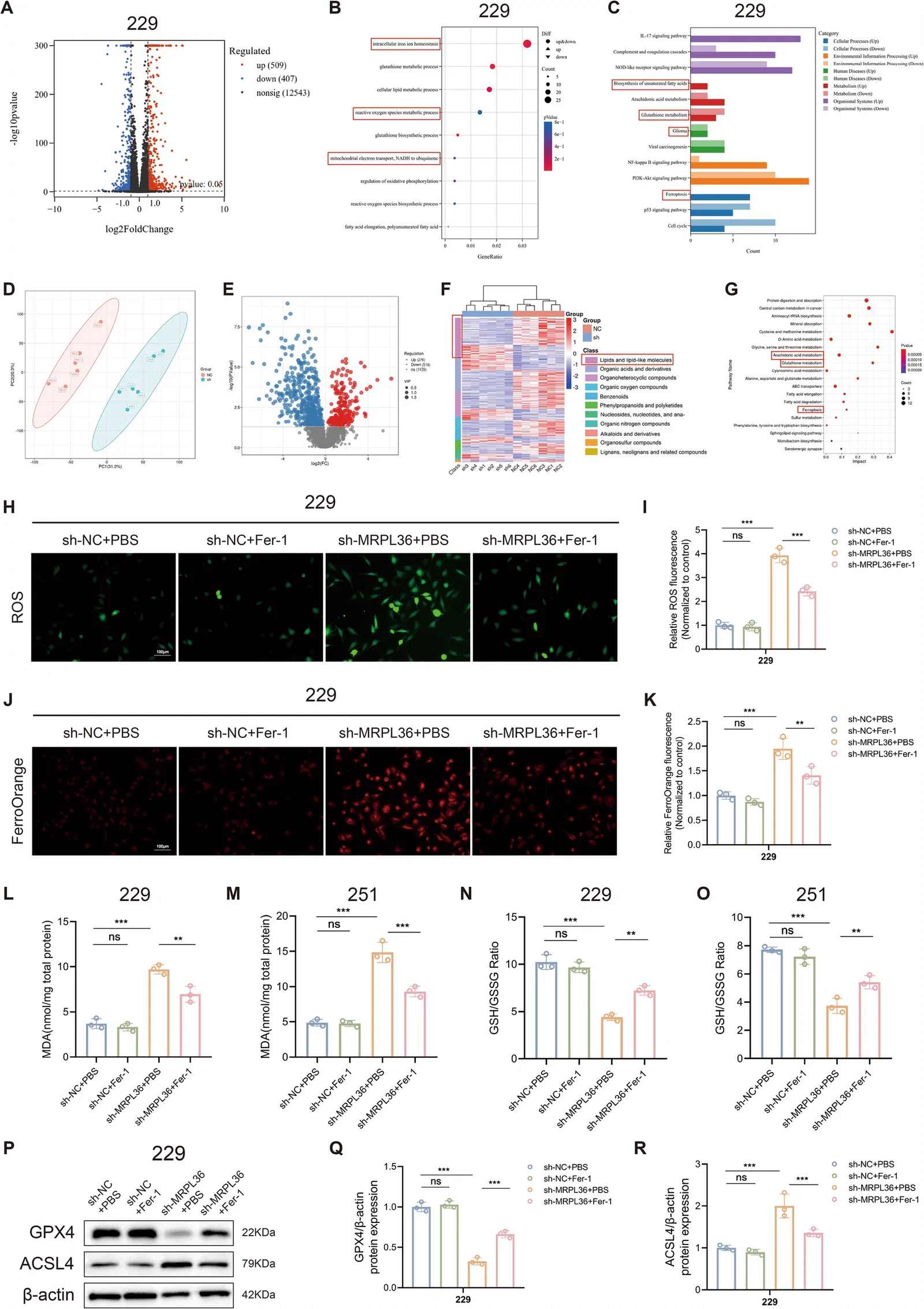
MRPL36 knockdown induces transcriptomic and metabolomic remodeling and ferroptosis in GBM cells (**A**) Volcano plot of differentially expressed genes between the control group and the MRPL36 knockdown group. (**B**-**C**) GO and KEGG enrichment analyses of differentially expressed genes. (**D**) PCA plot of non-targeted metabolomics data from the control and MRPL36 knockdown groups (*n* = 6). (**E**) Volcano plot of differential metabolites between the control and MRPL36 knockdown groups. (**F**) Cluster heatmap of differential metabolites. (**G**) KEGG enrichment analysis of differential metabolites. (**H**-**I**) ROS levels in LN229 cells: representative images (**H**) and quantitative analysis (**I**). (**J**-**K**) FerroOrange staining-mediated detection of intracellular Fe^2+^ levels in LN229 cells: representative images (**J**) and quantitative analysis (**K**). (**L**-**M**) Intracellular MDA levels in LN229 (**L**) and U251MG (**M**) cells. (**N**-**O**) Intracellular GSH/GSSG ratios in LN229 (**N**) and U251MG (**O**) cells. (**P**-**R**) Protein expression of GPX4 and ACSL4 in LN229 cells determined by Western blotting (**P**), with quantitative analysis of GPX4 (**Q**) and ACSL4 (**R**) expression levels. *P < 0.05, **P < 0.01, ***P < 0.001

Previous studies have confirmed that metabolic reprogramming and mitochondrial dysfunction are core characteristics of GBM, which can significantly drive tumor malignant progression and affect patient prognosis [27, 28]. To systematically elucidate the mechanism by which MRPL36 regulates the malignant progression of GBM, we first performed RNA sequencing on MRPL36 knockdown and control LN229 and U251MG cells. Differential gene expression analysis showed that MRPL36 knockdown upregulated a series of ferroptosis-related genes and downregulated multiple genes associated with cellular homeostasis and metabolic regulation (Fig. 3A, Supplementary Fig. 2A). GO enrichment analysis revealed that MRPL36 knockdown altered core biological processes linked to ferroptosis in two glioblastoma cell lines, including intracellular iron homeostasis, reactive oxygen species metabolism, lipid metabolism and glutathione metabolism. Further KEGG enrichment analysis confirmed that in addition to the canonical ferroptosis pathway, unsaturated fatty acid biosynthesis, fatty acid metabolism, glutathione metabolism and cell cycle-related pathways were also markedly enriched (Figs. 3B-C, Supplementary Fig. 2B-C). These results indicate that MRPL36 drives the malignant progression of glioblastoma mainly through regulating ferroptosis. It modulates cellular ferroptosis at the transcriptional level, and also regulates metabolic reprogramming and cell cycle homeostasis.

To further verify whether this regulation exists in the tumor microenvironment in vivo, we performed untargeted metabolomic analysis on tumor tissues from orthotopic xenograft mouse models. Principal component analysis (PCA) showed clear separation of metabolic profiles between groups (Fig. 3D), indicating that MRPL36 inhibition significantly alters the overall metabolic state of GBM. Further systematic analysis of differential metabolites demonstrated that lipids and lipid-like molecules exhibited the most significant changes after MRPL36 silencing (Figs. 3E-F). KEGG pathway enrichment analysis showed that these differential metabolites were significantly enriched in pathways closely related to lipid peroxidation and ferroptosis, including arachidonic acid metabolism and glutathione metabolism (Fig. 3G). Combined transcriptomic and metabolomic analyses indicated that disordered lipid metabolism is a key upstream event in MRPL36-regulated ferroptosis. We hypothesized that MRPL36 modulates ferroptosis by remodeling lipid metabolic homeostasis, thereby affecting the survival of GBM cells.

Next, to verify whether MRPL36 knockdown induces ferroptosis in GBM cells, we performed a series of functional assays. The levels of ROS, Fe^2+^, MDA, and the ratio of GSH to GSSG in LN229 and U251MG cells were measured to evaluate cellular oxidative stress, ferrous iron accumulation, lipid peroxidation, and antioxidant system changes. The results showed that compared with the negative control group, the MRPL36 knockdown group exhibited significantly increased levels of ROS, Fe^2+^, and MDA, and a markedly decreased GSH/GSSG ratio. These changes were significantly reversed by treatment with the specific ferroptosis inhibitor Ferrostatin-1 (Fer-1), whereas Fer-1 had no obvious effect on the negative control group (Figs. 3H-O, Supplementary Fig. 2E-H). Meanwhile, Western blot analysis showed that MRPL36 silencing significantly upregulated ACSL4 and downregulated GPX4 expression, and these effects were also reversed by Fer-1 (Figs. 3P-R; Supplementary Fig. 2I-K). Collectively, these results indicate that MRPL36 knockdown induces ferroptosis in GBM cells through modulating ferroptosis-associated metabolism and key protein expression, highlighting MRPL36 as a critical regulator in the ferroptosis network of GBM cells.

**Fig. 4:**
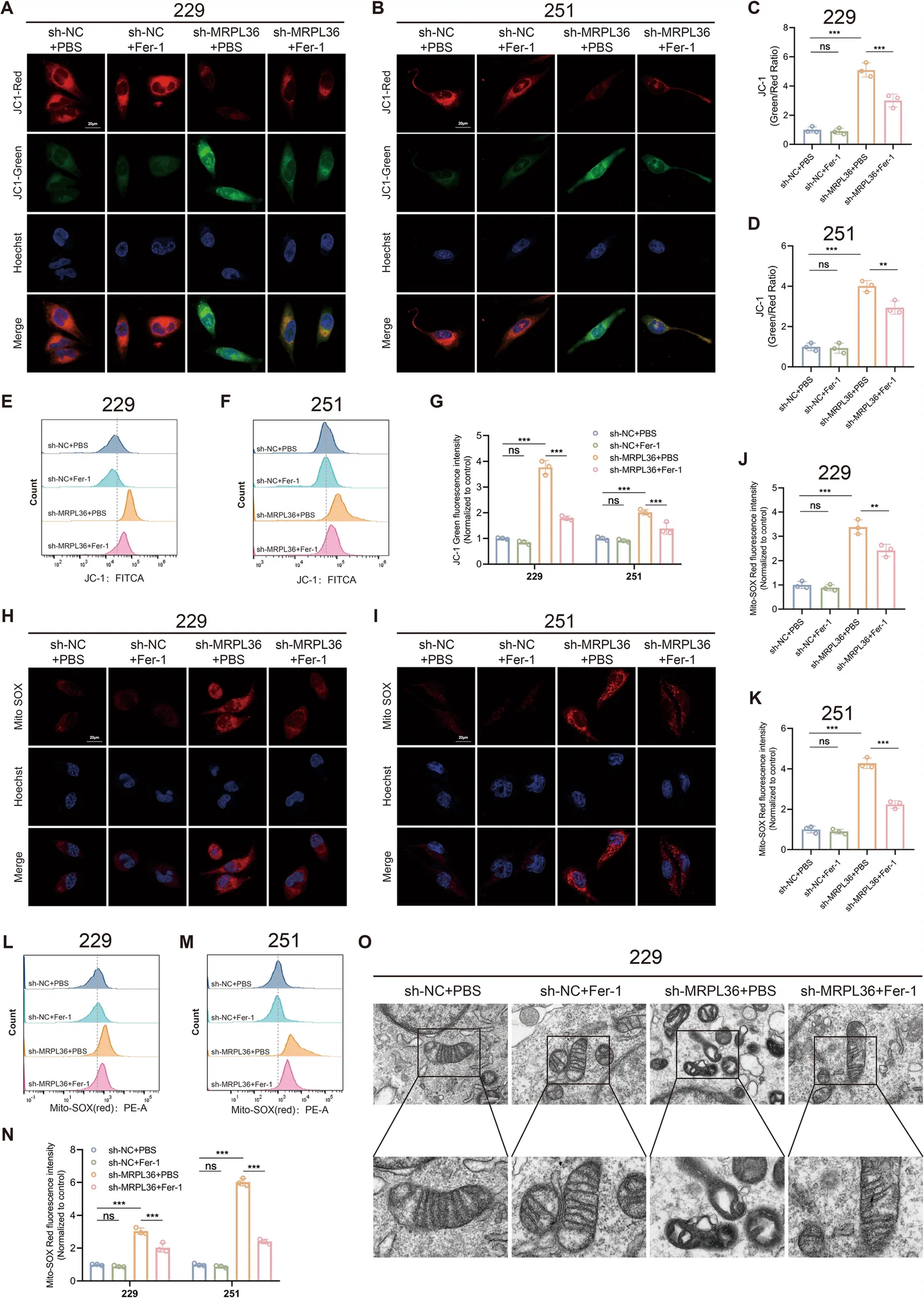
MRPL36 knockdown induces mitochondrial dysfunction in GBM cells (**A**-**D**) JC-1 fluorescence probe assay in LN229 (**A**, **C**) and U251MG (**B**, **D**) cells: representative images (**A**, **B**) and quantitative analysis (**C**, **D**). (**E**-**G**) Flow cytometric detection of JC-1 fluorescence signals in LN229 (**E**) and U251MG (**F**) cells, with quantitative analysis (**G**). (**H**-**K**) MitoSOX fluorescence probe assay in LN229 (**H**, **J**) and U251MG (**I**, **K**) cells: representative images (**H**, **I**) and quantitative analysis (**J**, **K**). (**L**-**N**) Flow cytometric detection of MitoSOX fluorescence signals in LN229 (**L**) and U251MG (**M**) cells, with quantitative analysis (**N**). (**O**) Transmission electron microscopy images of mitochondrial ultrastructure in LN229 cells. *P < 0.05, **P < 0.01, ***P < 0.001

Numerous studies have demonstrated that mitochondrial ribosomal proteins participate in the regulation of energy metabolism and ferroptosis in tumor cells by maintaining mitochondrial structural and functional homeostasis [29, 30]. As a key component of the large mitochondrial ribosomal subunit, aberrant expression of MRPL36 directly affects mitochondrial translation efficiency and is involved in multiple pathophysiological processes [8]. Therefore, we next investigated the role of MRPL36 in mitochondrial function in GBM cells. JC-1 fluorescence probe results showed that compared with the negative control group, the mitochondrial membrane potential (ΔΨm) was significantly decreased in the MRPL36 knockdown group. Fer-1 intervention partially reversed this effect but had no obvious influence on the negative control group (Figs. 4A-D). JC-1 flow cytometry analysis further confirmed that MRPL36 silencing significantly increased the proportion of JC-1 green fluorescence, which was partially reversed by Fer-1 treatment. This partial rescue suggests that while MRPL36 directly regulates mitochondrial function due to its mitochondrial localization, a portion of the observed mitochondrial alterations may occur as secondary events associated with ferroptosis (Figs. 4E-G). MitoSOX fluorescence probe results showed that the fluorescence signal was significantly enhanced in the MRPL36 knockdown group, indicating increased mitochondrial superoxide production. Fer-1 could markedly mitigate this elevated oxidative stress by alleviating secondary damage induced by ferroptosis, while exerting no apparent effects on the negative control group (Figs. 4H-K). Flow cytometry results were consistent with immunofluorescence staining (Figs. 4L-N). For morphological validation, we observed mitochondrial ultrastructure using transmission electron microscopy (TEM). Compared with the negative control group, mitochondria in the MRPL36 knockdown group exhibited typical ferroptosis-related abnormalities, including swelling, broken and sparse cristae, damaged membrane structure, and partial vacuolization. Fer-1 intervention markedly ameliorated these abnormalities (Fig. 4O, Supplementary Fig. 3A). These electron microscopic findings were consistent with JC-1 and MitoSOX staining results, further demonstrating that mitochondrial functional and morphological abnormalities following MRPL36 knockdown are closely linked to ferroptosis.

To explore whether ferroptosis mediates the regulatory effect of MRPL36 on the tumor phenotypes of GBM cells, we treated LN229 and U251MG cells with Fer-1 and examined its effects on proliferation, migration, and invasion in MRPL36-deficient cells. Colony formation and EdU assays showed that MRPL36 knockdown significantly suppressed cell colony formation and proliferation, and these effects were partially reversed by Fer-1 treatment, with no obvious influence on the negative control group (Supplementary Fig. 3B-I). Wound-healing and Transwell assays demonstrated that MRPL36 silencing also significantly inhibited cell migration and invasion, and this effect was effectively reversed by Fer-1, whereas Fer-1 had no obvious effect on the negative control group (Supplementary Fig. 3J-Q). In conclusion, these findings demonstrate that MRPL36 knockdown promotes ferroptosis and subsequently induces secondary mitochondrial dysfunction in GBM cells, leading to the suppression of cell proliferation, migration and invasion. Collectively, our results reveal that downregulation of MRPL36 triggers ferroptosis, thereby retarding the malignant progression of glioblastoma.

### MRPL36 stabilizes GPX4 by antagonizing its ubiquitination and degradation *via* C66 palmitoylation

After confirming that MRPL36 drives the malignant progression of GBM by regulating ferroptosis and mitochondrial function, we next investigated the underlying molecular mechanism. Accumulating evidence has verified that GPX4 serves as a core rate-limiting enzyme in ferroptosis and a key molecule maintaining mitochondrial oxidative homeostasis and the malignant phenotype of tumors [31, 32]. We therefore focused on dissecting the specific mechanism by which MRPL36 regulates GPX4.

**Fig. 5:**
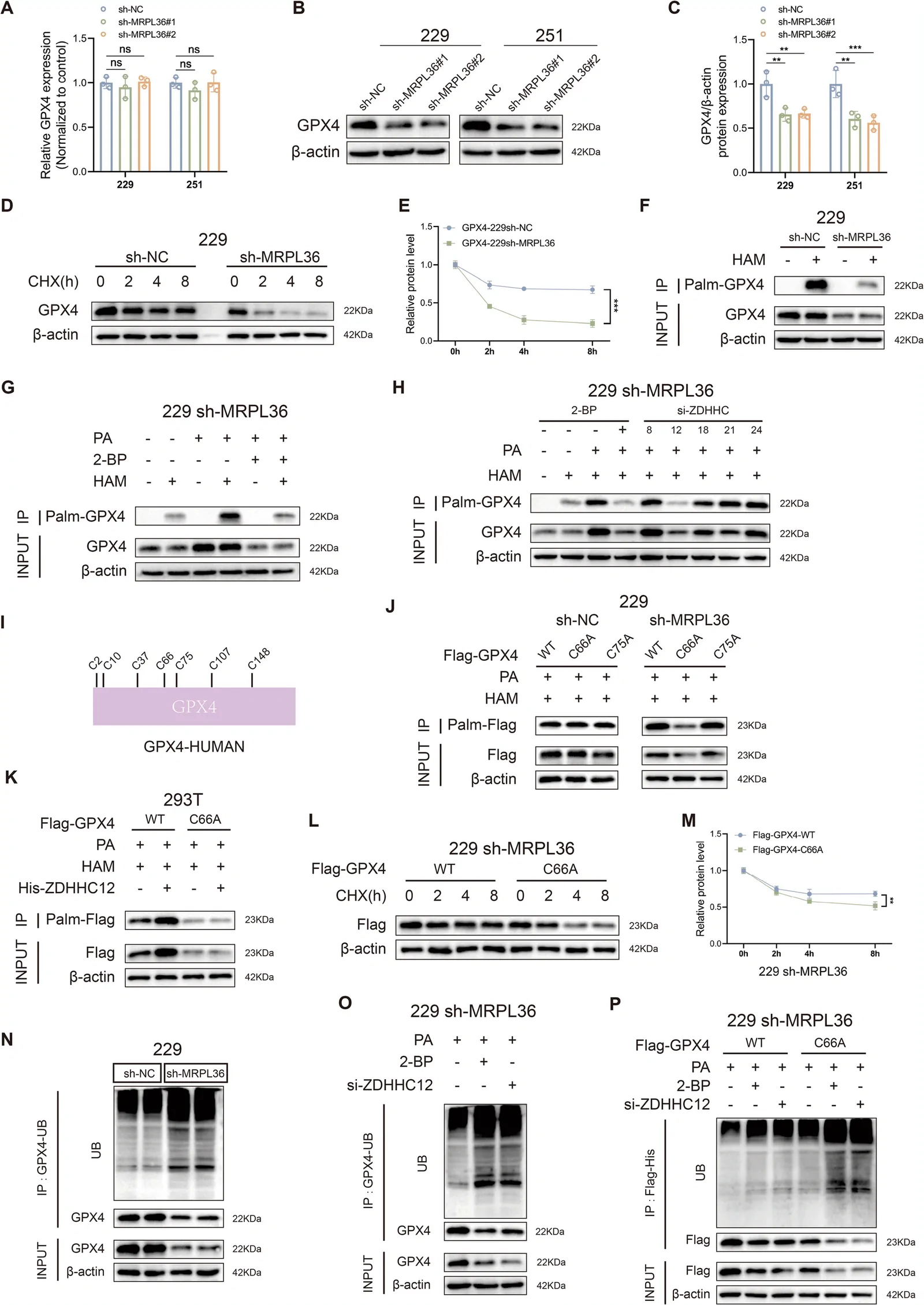
MRPL36 knockdown promotes GPX4 degradation by suppressing C66 palmitoylation in GBM cells (**A**) qRT-PCR analysis of GPX4 mRNA expression in LN229 and U251MG cells following sh-MRPL36 treatment. (**B**-**C**) Western blotting analysis of GPX4 protein expression in LN229 and U251MG cells after sh-MRPL36 treatment. (**D**-**E**) CHX chase assay (2.5 µg/mL CHX) showing GPX4 protein stability in sh-MRPL36-treated LN229 cells at 0, 4, and 8 h. (**F**) ABE assay for GPX4 palmitoylation levels in sh-NC and sh-MRPL36 LN229 cells after HAM treatment. (**G**) ABE assay detecting GPX4 palmitoylation in sh-MRPL36 LN229 cells following treatment with PA, 2-BP and HAM under six combined regimens. (**H**) ABE assay assessing GPX4 palmitoylation in sh-MRPL36 LN229 cells treated with PA, HAM, 2-BP, or ZDHHC knockdown. (**I**) Consensus sequence of GPX4 showing seven conserved cysteine residues across species. (**J**) ABE assay identifying GPX4 palmitoylation at the C66 and C75 sites. (**K**) ABE assay detecting GPX4 palmitoylation at the C66 site following ZDHHC12 treatment. (**L**-**M**) CHX chase assay evaluating GPX4 protein stability following palmitoylation at the C66 site. (**N**) Co-IP assay measuring GPX4 ubiquitination levels after sh-MRPL36 treatment. (**O**) Co-IP assay detecting GPX4 ubiquitination in MRPL36-silenced LN229 cells after 2-BP treatment or ZDHHC12 knockdown. (**P**) Co-IP assay for GPX4 ubiquitination in MRPL36-silenced LN229 cells expressing Flag-GPX4 (WT or C66A) after PA, 2-BP, or si-ZDHHC12 treatment. *P < 0.05, **P < 0.01, ***P < 0.001

First, we examined the expression changes of GPX4 at the transcriptional and translational levels. GPX4 mRNA levels did not differ significantly following MRPL36 knockdown, whereas its protein expression was markedly downregulated, suggesting that MRPL36 may regulate GPX4 stability at the post-translational level **(**Figs. 5A-C). To verify this hypothesis, we performed cycloheximide (CHX) chase assays in LN229 and U251MG cells. The results demonstrated that GPX4 protein degradation was significantly accelerated and the remaining protein level was lower at the same time points after MRPL36 silencing, confirming that reduced MRPL36 expression promotes GPX4 degradation and decreases its stability (Figs. 5D-E, Supplementary Fig. 4A-B). Combined with our previous untargeted metabolomics analysis, we found that MRPL36 knockdown significantly increased the content of palmitoyl-CoA (Supplementary Fig. 2D). As a key acyl donor for protein S-palmitoylation, the level of palmitoyl-CoA is often closely associated with protein palmitoylation. Accordingly, we hypothesized that the alteration in palmitoyl-CoA induced by MRPL36 depletion may regulate GPX4 protein stability by affecting its palmitoylation. We therefore further detected the palmitoylation level of GPX4. The results showed that MRPL36 inhibition significantly reduced GPX4 palmitoylation (Fig. 5F, Supplementary Fig. 4C). This result suggests that the global level of palmitoyl-CoA is not simply positively correlated with protein palmitoylation. Although MRPL36 inhibition elevated palmitoyl-CoA levels, mitochondrial dysfunction may ultimately reduce GPX4 palmitoylation by altering the modification microenvironment or enzyme activity.

To achieve a more robust detection of GPX4 palmitoylation, MRPL36-silenced LN229 and U251MG cells were supplemented with 50 µM exogenous palmitic acid (PA) for 6 h, a condition that did not significantly affect cell viability (Supplementary Fig. 4D-E). Under this PA-supplemented condition, the restoration of GPX4 palmitoylation in MRPL36-knockdown cells was readily detected. Conversely, co-treatment with the palmitoylation inhibitor 2-bromopalmitate (2-BP) markedly decreased GPX4 palmitoylation compared with PA treatment alone (Fig. 5G, Supplementary Fig. 4F). These results indicate that palmitoylation is a key process in maintaining GPX4 protein stability, and MRPL36 can regulate GPX4 stability by modulating its palmitoylation. Studies have shown that protein palmitoylation is mainly catalyzed by the zinc finger DHHC-type palmitoyltransferase (ZDHHC) family, and the palmitoylation of different substrate proteins is often specifically mediated by distinct ZDHHC members [33–35]. Based on this, we further screened and identified the key ZDHHC family member responsible for GPX4 palmitoylation. We performed survival prognosis analysis of 24 ZDHHC members using the SangerBox database and selected the top 5 genes with the most significant prognostic differences (Supplementary Fig. 4G-K). We then knocked down these five candidate ZDHHC proteins (ZDHHC8, 12, 18, 21, and 24) separately in MRPL36-downregulated GBM cells and detected GPX4 palmitoylation. The results showed that ZDHHC12 knockdown led to the most significant reduction in GPX4 palmitoylation, suggesting that ZDHHC12 may be the key regulatory molecule mediating GPX4 palmitoylation (Fig. 5H, Supplementary Fig. 4L).

To further identify the key site of GPX4 palmitoylation, we performed conservation analysis of its protein sequence and found seven highly conserved cysteine residues across species (Fig. 5I). According to previous studies, GPX4 palmitoylation mainly occurs at the C66 and C75 sites. We therefore focused on these two sites and constructed mutant plasmids [36, 37]. The results showed that in MRPL36 knockdown cells, C66 mutation significantly reduced GPX4 palmitoylation, whereas C75 mutation had no obvious effect. In negative control cells, neither mutation significantly altered GPX4 palmitoylation, indicating that C66 is the key palmitoylation site of GPX4 responsible for MRPL36-mediated regulation (Fig. 5J, Supplementary Fig. 4M). Based on the above results, we speculate that MRPL36 preferentially modulates GPX4 palmitoylation at the C66 site, while exerting negligible regulatory effects on C75 modification. In negative control cells with normal endogenous MRPL36 expression, GPX4 palmitoylation maintains its intrinsic steady state, and single mutation of either C66 or C75 fails to perturb the overall modification level. Upon MRPL36 knockdown, this homeostatic balance is disrupted, rendering GPX4 palmitoylation strongly dependent on the structural integrity of the C66 site; consequently, C66 mutation markedly diminishes GPX4 palmitoylation. In contrast, C75 palmitoylation is insensitive to changes in MRPL36 expression. Accordingly, C75 mutation has no obvious impact on GPX4 palmitoylation irrespective of MRPL36 knockdown. Exogenous experiments in 293T cells confirmed that C66 mutation (C66A) significantly reduced the palmitoylation level of Flag-GPX4. Overexpression of His-ZDHHC12 markedly enhanced the palmitoylation of wild-type Flag-GPX4 but had no obvious effect on the C66A (Fig. 5K). These results indicate that ZDHHC12 mediates GPX4 palmitoylation mainly through the C66 site.

To explore the regulatory role of C66 palmitoylation in GPX4 protein stability, we knocked down MRPL36 in LN229 and U251MG cells, transfected Flag-GPX4-WT and Flag-GPX4-C66A plasmids respectively, and performed CHX chase assays. The results showed that GPX4 degradation was significantly faster in the C66A group than in the wild-type group, with lower protein expression at the same time points (Figs. 5L-M, Supplementary Fig. 4N-O). Given that protein degradation mainly depends on the ubiquitin-proteasome pathway and that different post-translational modifications of the same protein are cross-regulated [38, 39], we hypothesized that C66 palmitoylation maintains GPX4 stability by antagonizing ubiquitination, with a competitive inhibitory relationship between these two modifications. Further experiments showed that MRPL36 silencing significantly increased GPX4 ubiquitination and decreased its protein expression, suggesting that MRPL36 maintains GPX4 stability by negatively regulating its ubiquitin-dependent degradation (Fig. 5N, Supplementary Fig. 4P). In MRPL36 knockdown cells, inhibition of palmitoylation with 2-BP or knockdown of ZDHHC12 further upregulated GPX4 ubiquitination and accelerated protein degradation (Fig. 5O, Supplementary Fig. 4Q). Meanwhile, site mutation and palmitoylation intervention experiments in MRPL36 knockdown cells showed that under the same treatment conditions, GPX4 ubiquitination was significantly higher in the C66A group than in the wild-type group, whereas protein expression showed the opposite trend (Fig. 5P, Supplementary Fig. 4R). These results further confirm the competitive inhibitory relationship between GPX4 palmitoylation and ubiquitination.

### MRPL36 regulates TRIM33 to promote GPX4 degradation *via* K48-linked ubiquitination and regulate GBM progression

**Fig. 6:**
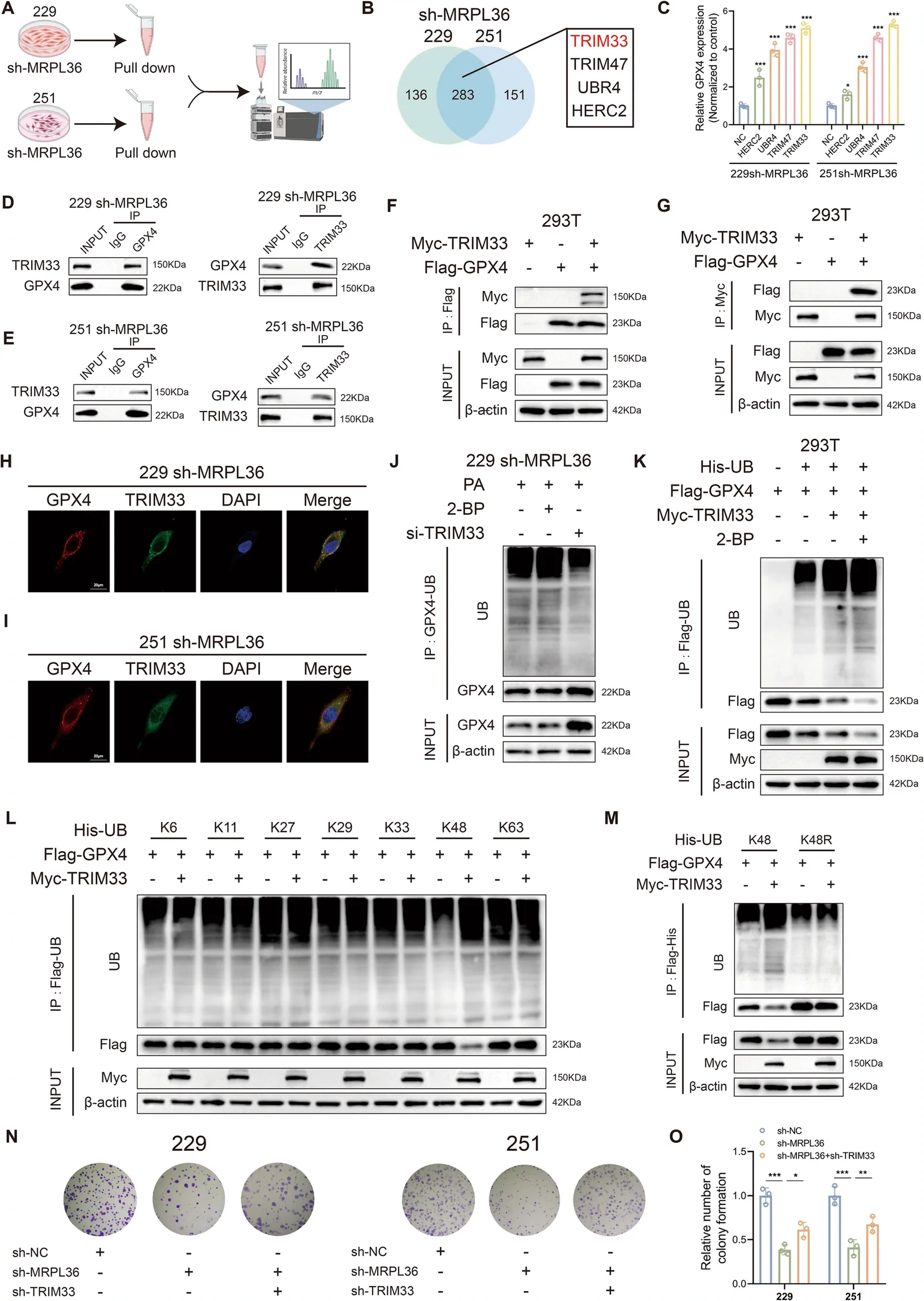
MRPL36 knockdown promotes TRIM33-dependent ubiquitination and degradation of GPX4 to drive ferroptosis in GBM cells (**A**) Pull-down assay coupled with LC-MS analysis for GPX4-interacting proteins in sh-MRPL36-treated LN229 and U251MG cells. (**B**) Mass spectrometry results suggested a potential interaction between GPX4 and TRIM33. (**C**) Western blotting analysis of GPX4 protein expression in sh-MRPL36-treated LN229 and U251MG cells after TRIM33 knockdown. (**D**-**E**) Co-IP assay for the interaction between GPX4 and TRIM33 in sh-MRPL36-treated LN229 and U251MG cells. (**F**-**G**) Co-IP assay for the interaction between GPX4 and TRIM33 in 293T cells co-transfected with Myc-TRIM33 and Flag-GPX4 plasmids. (**H**-**I**) Immunofluorescence staining for subcellular colocalization of GPX4 and TRIM33 in sh-MRPL36-treated LN229 (**H**) and U251MG (**I**) cells. (**J**) GPX4 ubiquitination levels in sh-MRPL36-treated LN229 cells after PA, 2-BP, or si-TRIM33 treatment detected by Co-IP. (**K**) Co-IP assay for Flag-GPX4 ubiquitination in 293T cells co-transfected with Flag-GPX4, Myc-TRIM33, and His-UB after 2-BP treatment. (**L**) Co-IP assay for GPX4 ubiquitination in 293T cells co-transfected with Flag-GPX4, Myc-TRIM33, and His-UB with different lysine site mutations (K6, K11, K27, K29, K33, K48, K63). (**M**) Co-IP assay for GPX4 ubiquitination in 293T cells co-transfected with Flag-GPX4, Myc-TRIM33, and His-UB (K48) or His-UB (K48R). (**N**-**O**) Colony formation assay in LN229 and U251MG cells after MRPL36 knockdown alone or combined with TRIM33 knockdown. *P < 0.05, **P < 0.01, ***P < 0.001

Based on the competitive inhibitory relationship between GPX4 palmitoylation and ubiquitination, we used MRPL36-knockdown LN229 and U251MG cells to screen for ubiquitin ligases that interact with GPX4 (Fig. 6A). According to the interacting protein profile obtained by IP-MS, we identified four ubiquitin ligases potentially interacting with GPX4 (TRIM33, TRIM47, UBR4, and HERC2), among which TRIM33 showed the highest abundance (Fig. 6B). We further silenced these four candidate ubiquitin ligases separately in MRPL36-knockdown GBM cells. The results showed that GPX4 protein expression was most significantly increased after TRIM33 knockdown, suggesting that TRIM33 is the key ubiquitin ligase mediating GPX4 ubiquitination and degradation (Fig. 6C, Supplementary Fig. 5A-B). To directly assess whether TRIM33 regulates GPX4 ubiquitination, we performed Co-IP assays to detect GPX4 ubiquitination levels following TRIM33 knockdown. The results demonstrated that TRIM33 silencing markedly reduced GPX4 ubiquitination in both LN229 and U251MG cells (Supplementary Fig. 5C-D). These findings collectively indicate that TRIM33 acts as an E3 ubiquitin ligase to mediate GPX4 ubiquitination and degradation. In addition, Western blot analysis revealed that GPX4 interacted with TRIM33 in MRPL36-knockdown LN229, U251MG, and 293T cells (Figs. 6D-G). Immunofluorescence co-localization assays further confirmed obvious co-localization of these two proteins in MRPL36-silenced LN229 and U251MG cells (Figs. 6H-I). These results collectively indicate that GPX4 directly interacts with TRIM33.

Next, we further validated the regulatory role of TRIM33 in GPX4 ubiquitination. In MRPL36-knockdown cells, TRIM33 interference reduced GPX4 ubiquitination and upregulated GPX4 protein expression (Figs. 6J, Supplementary Fig. 5E). In control LN229 and U251MG cells, treatment with the palmitoylation inhibitor 2-BP increased GPX4 ubiquitination, whereas TRIM33 silencing decreased GPX4 ubiquitination compared with untreated controls (Supplementary Fig. 5F-G). To further verify the regulatory effect of TRIM33 on GPX4 ubiquitination, we established a gradient intervention system in 293T cells: Flag-GPX4, His-Ub, and Myc-TRIM33 were co-transfected in sequence, supplemented with 2-BP treatment. The results showed that with the introduction of TRIM33 and 2-BP intervention, GPX4 ubiquitination was gradually increased, while Flag-GPX4 protein expression was gradually decreased, suggesting that TRIM33 promotes GPX4 ubiquitination and degradation, and inhibition of palmitoylation further enhances this effect (Fig. 6K).

The linkage type of polyubiquitin chains determines their spatial conformation and downstream regulatory function, and different lysine residues on ubiquitin can assemble ubiquitin chains with distinct functions [40, 41]. To identify the ubiquitin chain type catalyzed by TRIM33 on GPX4, we performed ubiquitin chain typing assays in 293T cells. A panel of single lysine-mutated His-Ub plasmids covering major linkage sites (K6, K11, K27, K29, K33, K48, K63) were co-transfected with Flag-GPX4, with or without Myc-TRIM33. Ubiquitin chains conjugated to GPX4 were enriched *via* anti-Flag co-immunoprecipitation. The results revealed that TRIM33 specifically enhanced K48-linked ubiquitination of GPX4, whereas no alterations were observed for the well-characterized K11, K63 and other chain types (K6, K27, K29, K33) (Fig. 6L). To further verify the critical role of the K48 site in TRIM33-driven GPX4 degradation, we co-transfected 293T cells with Flag-GPX4, Myc-TRIM33, plus either wild-type His-Ub or the K48-inactivating mutant (K48R). Relative to the K48R mutant, wild-type ubiquitin increased K48-linked polyubiquitination and reduced GPX4 protein abundance (Fig. 6M). Collectively, these data demonstrate that TRIM33 mediates K48-linked polyubiquitination of GPX4 to facilitate its proteasomal degradation.

Subsequently, we performed functional assays to explore whether TRIM33 is involved in regulating GBM progression. Colony formation and EdU assays showed that MRPL36 knockdown significantly inhibited cell proliferation in LN229 and U251MG cells, whereas combined TRIM33 knockdown partially reversed this effect (Figs. 6N-O, Supplementary Fig. 6A-B). Wound-healing and Transwell assays demonstrated that MRPL36 silencing markedly suppressed migration and invasion of LN229 and U251MG cells, while combined TRIM33 knockdown partially restored these phenotypes (Supplementary Fig. 6C-F). These results indicate that TRIM33 interference partially reverses the reduction in GBM cell migration and invasion caused by MRPL36 knockdown.

### MRPL36 promotes GBM growth in vivo by inhibiting ferroptosis

**Fig. 7:**
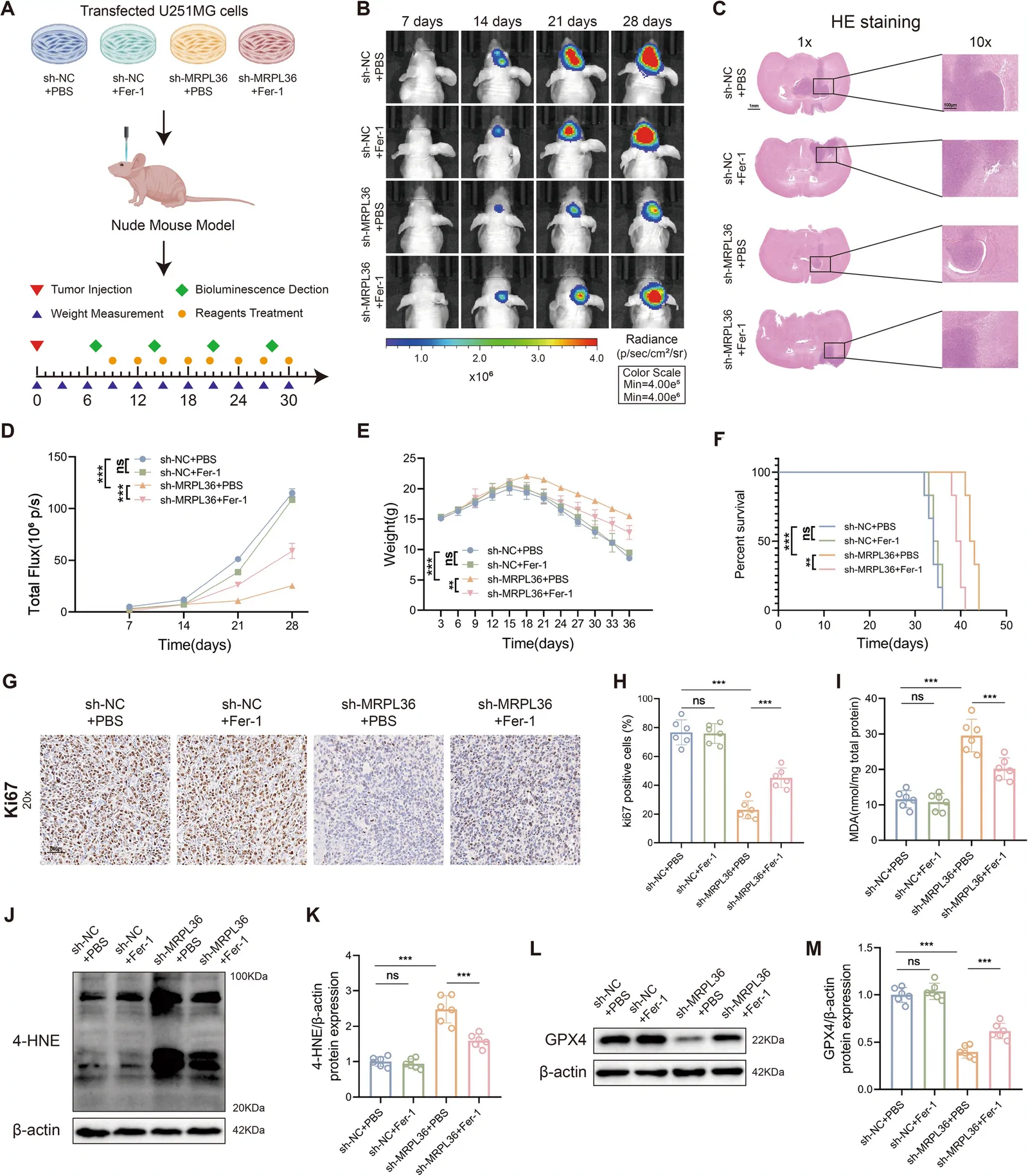
MRPL36 knockdown suppresses GBM tumor growth and induces ferroptosis in vivo (**A**) Establishment of a mouse model of intracranial xenograft tumor. (**B**-**C**) In vitro imaging combined with HE staining was used to evaluate the fluorescence intensity and volume of intracranial tumors in nude mice of each group. (**D**) Line graph comparing the temporal change trend of total fluorescence intensity of intracranial tumors in nude mice of each group. (**E**) Line graph showing the body weight changes and intergroup differences of nude mice in each group at different time points. (**F**) Survival analysis of tumor-bearing mice in each group. (**G**-**H**) IHC detection and analysis of the expression difference of Ki67 in tumor tissues of nude mice in each group. (I) MDA expression levels in tumor tissues of nude mice in each group. (**J**-**K**) Western blotting analysis of 4-HNE expression in tumor tissues of nude mice in each group. (**L**-**M**) Western blotting analysis of GPX4 expression in tumor tissues of nude mice in each group. *P < 0.05, **P < 0.01, ***P < 0.001

Given the critical role of MRPL36 in suppressing ferroptosis and maintaining mitochondrial homeostasis to drive GBM progression, we established an in vivo model with four groups: sh-NC + PBS, sh-NC + Fer-1, sh-MRPL36 + PBS, and sh-MRPL36 + Fer-1, to investigate whether the ferroptosis inhibitor Fer-1 could reverse MRPL36 knockdown-mediated GBM progression (Fig. 7A). In immunodeficient nude mouse models, tumor growth in the sh-MRPL36 + PBS group was significantly suppressed compared with the sh-NC + PBS group. Following Fer-1 treatment, tumor growth in the sh-MRPL36 + Fer-1 group was significantly increased compared with the sh-MRPL36 + PBS group, but remained lower than that in the sh-NC + PBS group. No significant difference was observed between the two sh-NC groups. Tumor fluorescence intensity followed the same pattern (Figs. 7B-D).

Furthermore, compared with the sh-MRPL36 + PBS group, Fer-1 treatment significantly alleviated body weight loss and prolonged survival in MRPL36-knockdown tumor-bearing mice (sh-MRPL36 + Fer-1 group) (Figs. 7E-F). In addition, immunohistochemical analysis revealed that Fer-1 treatment markedly upregulated the expression of the proliferation marker Ki-67 in tumor tissues of the sh-MRPL36 + Fer-1 group (Figs. 7G-H). To clarify the regulatory effect of MRPL36 depletion on ferroptosis in vivo, we examined relevant biomarkers in local tumor tissues. Compared with the sh-NC + PBS group, the sh-MRPL36 + PBS group exhibited markedly upregulated levels of the lipid peroxidation markers MDA (Figs. 7I) and 4-HNE (Figs. 7J-K), along with reduced expression of the core ferroptosis regulator GPX4 (Figs. 7L-M). In the sh-MRPL36 + Fer-1 group, these alterations were partially rescued by Fer-1 treatment. In contrast, Fer-1 administration had no significant effect on these biomarkers in either of the sh-NC groups. Collectively, these results demonstrate that Fer-1 acts predominantly at the tumor site to antagonize MRPL36 knockdown-induced ferroptosis, thereby partially reversing MRPL36 knockdown-mediated GBM progression in vivo.

## Discussion

**Fig. 8:**
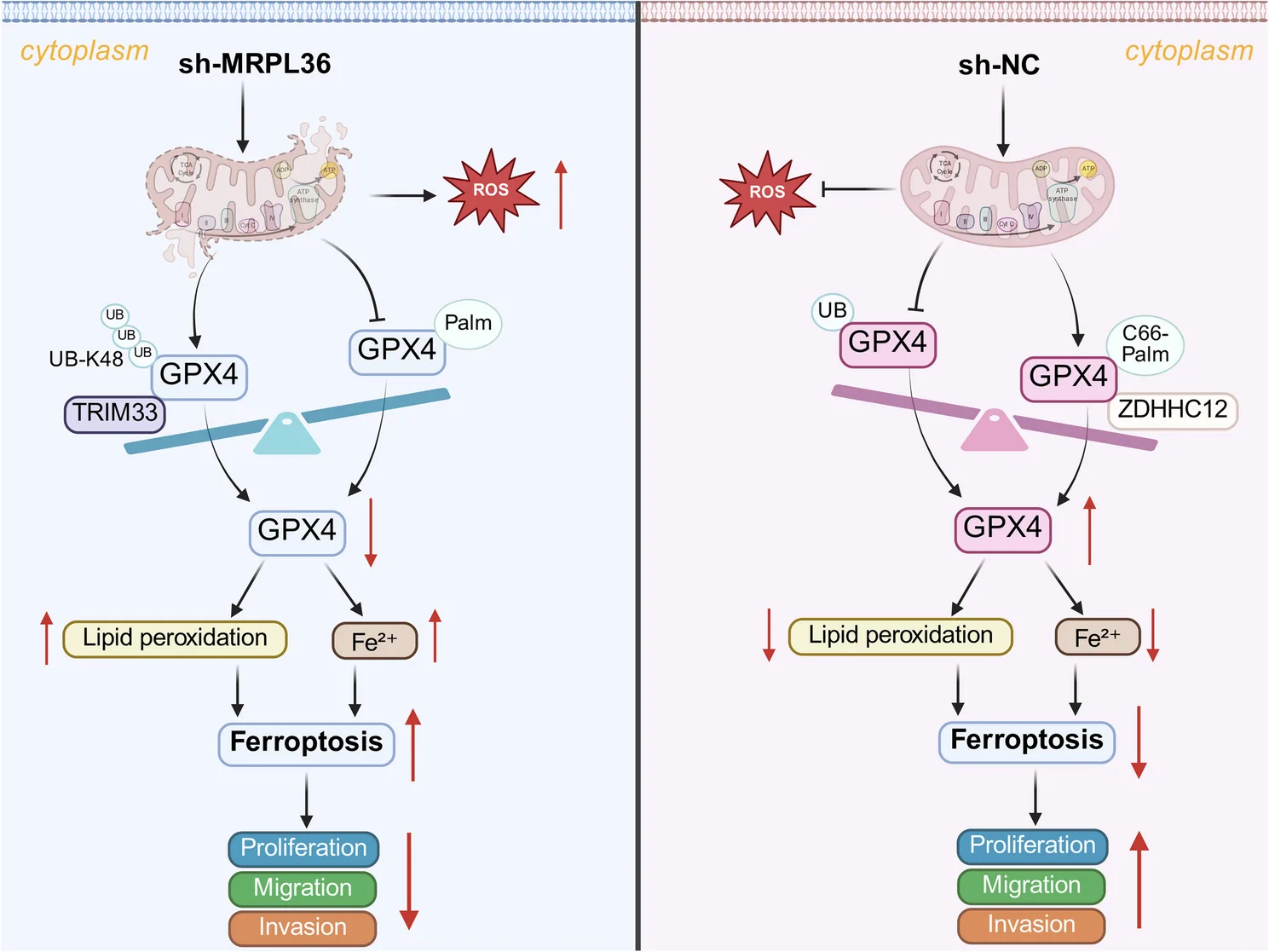
Schematic illustration of the molecular mechanism in this study

This study systematically clarified the core role and molecular mechanism of mitochondrial ribosomal protein MRPL36 in the malignant progression of GBM. It was found that MRPL36, as a key upstream regulatory factor, is involved in ZDHHC12-mediated GPX4 palmitoylation at the C66 site through reshaping mitochondrial functional homeostasis and lipid metabolism reprogramming. This specific palmitoylation modification can effectively antagonize the K48-linked polyubiquitination and proteasomal degradation of GPX4 mediated by TRIM33, thereby maintaining the stability of GPX4 protein and inhibiting the occurrence of ferroptosis (Fig. 8). By integrating multiple approaches including tumor tissue immunohistochemistry, cellular functional assays, mitochondrial function analysis, RNA-seq, and untargeted metabolomics, we not only verified the pivotal role of MRPL36 in GBM progression but also established the MRPL36-ZDHHC12/TRIM33-GPX4 axis as a critical hub connecting mitochondrial regulation, protein post-translational modifications, and the ferroptosis pathway. These findings highlight the key regulatory function of MRPL36 in GBM and provide a novel therapeutic strategy and theoretical basis for targeting the ZDHHC12 or TRIM33-mediated crosstalk between GPX4 palmitoylation and ubiquitination to stabilize GPX4 and inhibit ferroptosis, as well as new insights for the mechanistic research and precision targeted therapy of GBM.

As a key component of the large subunit of the mitochondrial ribosome, the core functional positioning of MRPL36 is directly associated with the regulation of mitochondrial homeostasis, and its abnormal expression will inevitably trigger a chain reaction of mitochondrial function, which is the key regulatory node revealed in this study [8]. Compared with other MRPL family members, MRPL36 exhibits unique functional specificity in regulating mitochondrial homeostasis. Previous studies have shown that MRPL36 contains a mitochondria-specific C-terminal domain critical for nascent protein assembly and its own stability, and that it determines the rate of respiratory chain assembly by stabilizing ribosomal subunit interactions [8]. In the present study, we further revealed that MRPL36 plays a non-redundant role in suppressing ferroptosis in GBM cells, distinguishing it from other MRPL members, although whether these members have distinct or overlapping functions in GBM progression warrants further investigation. The experimental results of this study showed that the decrease in mitochondrial membrane potential, accumulation of superoxide, and damage to ultra-microstructure induced by MRPL36 knockdown are not simple structural abnormalities, but are caused by the impairment of its core function in regulating mitochondrial translation efficiency and maintaining mitochondrial functional homeostasis. The normal expression of MRPL36 can provide basic support for cellular metabolism by stabilizing mitochondrial structure and function, while its depletion will break the balance of mitochondrial function and further trigger downstream metabolic disorders. From the metabolic perspective, untargeted metabolomic experiments confirmed that the significant abnormalities in lipid metabolism induced by MRPL36 knockdown are essentially the external manifestation of the coordinated imbalance of energy metabolism and material metabolism after mitochondrial function impairment. This metabolic reprogramming not only changes the levels of key metabolites such as palmitic acid in cells but also indirectly affects the balance of post-translational modifications of GPX4, forming a chain regulatory network of “metabolic reprogramming and modification imbalance”, which also explains why MRPL36 depletion further exacerbates the occurrence of ferroptosis. Notably, Fer-1 partially alleviates only secondary mitochondrial dysfunction upon MRPL36 knockdown but fails to restore primary mitochondrial damage directly triggered by MRPL36 loss owing to its mitochondrial localization. This partial rescue indicates that part of mitochondrial dysfunction develops downstream of ferroptosis and can be relieved by Fer-1. Nonetheless, these findings further confirm the close association between mitochondrial dysfunction and ferroptosis, and suggest that MRPL36 acts as an important upstream regulatory factor by maintaining mitochondrial homeostasis. Its mechanism of action extends beyond simple “protein expression regulation” and involves the integrated processes of mitochondrial function, metabolic reprogramming, and ferroptosis regulation.

GPX4 is a core molecule in ferroptosis regulation, and its stability directly determines the sensitivity of cells to ferroptosis [42, 43]. A key finding of this study is that MRPL36 achieves precise regulation of GPX4 stability by modulating the balance between palmitoylation and ubiquitination of GPX4, which also reveals the key role of post-translational modifications in ferroptosis regulation [44, 45]. From the molecular mechanism, IP-MS screening and functional verification confirmed that TRIM33 is a specific ubiquitin ligase of GPX4, and the K48-linked polyubiquitination mediated by TRIM33 is the core signal for GPX4 degradation. In contrast, the palmitoylation of GPX4 mediated by ZDHHC12 may serve as an antagonistic signal against this ubiquitination degradation pathway. Palmitoylation modification not only enhances the structural stability of GPX4 itself but may also hinder the binding of TRIM33 to GPX4, potentially through changes in spatial conformation, thereby inhibiting the ubiquitination and degradation of GPX4. The experimental results showed that the decrease in palmitic acid level and the impairment of GPX4 palmitoylation caused by MRPL36 knockdown will lead to the enhancement of TRIM33-mediated K48-linked polyubiquitination of GPX4, which further confirms that MRPL36 plays a core mediating role in the regulatory process linking metabolism, post-translational modification, and protein stability. MRPL36 provides the necessary metabolic basis for GPX4 palmitoylation by maintaining mitochondrial function and lipid metabolic homeostasis, and then realizes precise regulation of GPX4 stability through the competitive balance between palmitoylation and ubiquitination. Direct evidence that palmitoylation and ubiquitination compete for the same GPX4 molecule is currently lacking. Nevertheless, several studies support competitive inhibition between these two modifications on the same protein. For example, palmitoylation of P16 at Cys72 suppresses its ubiquitination-mediated degradation [46], and PD-L1 palmitoylation blocks its ubiquitination and lysosomal degradation [47]. In the context of ferroptosis, FASN-mediated USP5 palmitoylation reduces GPX4 ubiquitination [42]. Collectively, these findings support an antagonistic relationship between palmitoylation and ubiquitination. Based on our data showing that inhibiting one modification enhances the other, we hypothesize that GPX4 palmitoylation and ubiquitination may exhibit competitive inhibition, potentially through spatial conformation changes or competition for the same or adjacent residues. Nonetheless, this “metabolism-modification” coordinated regulatory mode not only enriches our understanding of the regulatory mechanism of GPX4 but also provides a new target direction for the precise treatment of GBM, that is, inhibiting tumor progression by regulating this modification balance to achieve precise intervention of ferroptosis.

The MRPL36-ZDHHC12/TRIM33-GPX4 axis provides a new perspective on the link between mitochondrial function and post-translational modifications. Given their distinct subcellular localizations, with MRPL36 residing in the mitochondrial ribosome and ZDHHC12 located in the ER or Golgi apparatus, understanding how MRPL36 signals to ZDHHC12 represents an important direction for future investigation. We hypothesize that MRPL36 knockdown triggers mitochondrial oxidative stress, and the resulting ROS can diffuse into the cytoplasm, thereby indirectly affecting ZDHHC12-mediated GPX4 palmitoylation. Further exploration of this signaling cascade, such as screening for key transcription factors or kinases involved in the mitochondrial stress response, will help elucidate the underlying mechanism. Moreover, the observation that C66A, but not C75A, reduces GPX4 palmitoylation only upon MRPL36 knockdown suggests the presence of compensatory mechanisms in the basal state. Future studies, including double-mutant construction and CRISPR screening, are warranted to test these hypotheses. Additionally, future studies could employ targeted lipidomics and mass spectrometry-based approaches will be employed to quantitatively link specific lipid peroxides, such as arachidonic acid-containing phospholipids, to GPX4 modifications, thereby deepening our mechanistic understanding of how MRPL36-driven metabolic reprogramming modulates the GPX4 palmitoylation-ubiquitination balance.

The clinical translational potential of this study warrants further discussion. MRPL36, ZDHHC12, and TRIM33 may serve as novel prognostic biomarkers or therapeutic targets for GBM, as mitochondrial ribosomal proteins are emerging as promising clinical biomarkers closely associated with patient survival and treatment response in various cancers. Regarding therapeutic targeting, ZDHHC12-mediated palmitoylation could be intervened using palmitoyltransferase inhibitors, as exemplified by the ZDHHC inhibitor TTZ1 that restores ferroptosis sensitivity in prostate cancer [48]. Although ZDHHC12-specific inhibitors are not yet available, our findings provide a rationale for their development. For TRIM33, while no specific inhibitors have been reported, the TRIM family is increasingly recognized as druggable [49]. Accumulating evidence has established that ferroptosis plays a critical role in temozolomide resistance [50]. Given that MRPL36 functions as an upstream regulator of GPX4 palmitoylation, it is plausible that MRPL36 or ZDHHC12 expression levels may correlate with temozolomide responsiveness. Future clinical cohort studies are warranted to evaluate whether these targets can predict temozolomide sensitivity and to assess the translational potential of targeting this axis to overcome drug resistance.

In summary, this study systematically explored the regulatory mechanism of MRPL36 in GBM progression and ferroptosis, and clarified that MRPL36 acts as a key upstream regulatory hub in GBM development. MRPL36 maintains mitochondrial functional homeostasis and regulates lipid metabolism balance, thereby participating in ZDHHC12-mediated GPX4 palmitoylation at the C66 site, antagonizing TRIM33-mediated K48-linked polyubiquitination and degradation of GPX4, and ultimately inhibiting ferroptosis to promote GBM progression. In vivo experiments further confirmed that MRPL36 knockdown significantly inhibits tumor growth, while the ferroptosis inhibitor Fer-1 can reverse this inhibitory effect, indicating that the regulatory role of MRPL36 in GBM is closely related to ferroptosis regulation. This study reveals a novel mechanism by which MRPL36 regulates GBM progression through the “mitochondrial function-lipid metabolism-post-translational modification” regulatory axis. It also provides a new theoretical basis and therapeutic target for the precise treatment of GBM. These findings have significant implications for improving the diagnosis, treatment, and prognosis of GBM patients.

## Supplementary Information


Supplementary Material 1. Supplementary Material 1: Supplementary Fig. 1: (A) Construction of a nude mouse model of intracranial xenograft tumor. (B-C) Intracranial tumor fluorescence intensity and volume were observed and evaluated by in vivo imaging and HE staining. (D) A line chart was used to analyze the difference in total fluorescence intensity in nude mice between the control and sh-MRPL36 groups. (E) A line chart compared body weight changes in the two groups of nude mice at different time points. (F) Survival analysis was performed on tumor-bearing mice in both groups. (G-H) IHC was used to detect the differential expression of Ki67 in tumor tissues of the two groups. *P < 0.05, **P < 0.01, ***P < 0.001. Supplementary Fig. 2: (A) Volcano plot of differentially expressed genes between control and sh-MRPL36 groups. (B-C) GO/KEGG enrichment analyses showing significant changes in ferroptosis-related pathways. (D) Non-targeted metabolomics revealing differential expression of palmitoyl-CoA. (E-F) Representative ROS fluorescence images (E) and quantitative analysis (F) of four cell groups. (G-H) Representative Fe2+ fluorescence images (G) and quantitative analysis (H) of four cell groups. (I-K) Western blotting (I) and quantitative statistics (J, K) of GPX4 and ACSL4 expression in four cell groups. *P < 0.05, **P < 0.01, ***P < 0.001. Supplementary Fig. 3: (A) Transmission electron microscopy images of mitochondrial ultrastructure in U251MG cells. (B-E) Colony formation assay in control and MRPL36-knockdown LN229 and U251MG cells treated with PBS or Fer-1, with representative images (B, C) and quantitative bar graphs (D, E). (F-I) EdU proliferation assay in the aforementioned four groups of LN229 and U251MG cells, with representative fluorescence images (F, G) and statistical graphs (H, I). (J-M) Wound healing assay to evaluate the migratory ability of the aforementioned four groups of LN229 and U251MG cells, with representative images (J, K) and statistical graphs (L, M). (N-Q) Transwell assay of migratory and invasive abilities in the aforementioned four groups of LN229 and U251MG cells, with representative images (N, O) and quantitative bar graphs (P, Q). *P < 0.05, **P < 0.01, ***P < 0.001. Supplementary Fig. 4: (A-B) CHX chase assay (2.5 µg/mL CHX) for GPX4 protein stability in control and MRPL36-knockdown U251MG cells at 0, 2, 4, and 8 h: representative Western blotting images (A) and quantitative analysis (B). (C) ABE assay for GPX4 palmitoylation levels in control and MRPL36-knockdown U251MG cells with or without HAM treatment. (D-E) Relative cell viability of control and MRPL36-knockdown LN229 (D) and U251MG (E) cells treated with palmitic acid (0, 25, 50, 100 µM) for 6 h. (F) ABE assay for GPX4 palmitoylation in sh-MRPL36-treated U251MG cells after treatment with PA, 2-BP, and HAM as indicated. (G-K) Survival prognosis analysis of ZDHHC8 (G), ZDHHC12 (H), ZDHHC18 (I), ZDHHC21 (J), and ZDHHC24 (K) was conducted based on the SangerBox database. (L) ABE assay for GPX4 palmitoylation in sh-MRPL36-treated U251MG cells treated with PA, HAM, 2-BP, or si-ZDHHC (8, 12, 18, 21, 24) as indicated. (M) ABE assay for Flag-GPX4 palmitoylation in control and MRPL36-knockdown U251MG cells expressing wild-type (WT), C66A, or C75A Flag-GPX4 after PA and HAM treatment. (N-O) CHX chase assay (2.5 µg /mL CHX) for GPX4 protein stability in sh-MRPL36-treated U251MG cells expressing wild-type (WT) or C66A Flag-GPX4 at 0, 2, 4, and 8 h: representative Western blot images (N) and quantitative analysis (O). (P) Co-IP assay for GPX4 ubiquitination levels in control and MRPL36-knockdown U251MG cells. (Q) Co-IP assay for GPX4 ubiquitination levels in sh-MRPL36-treated U251MG cells after treatment with PA, 2-BP, or si-ZDHHC12. (R) Co-IP assay for GPX4 ubiquitination in sh-MRPL36-treated U251MG cells expressing wild-type (WT) or C66A Flag-GPX4 after treatment with PA, 2-BP, or si-ZDHHC12 as indicated. *P < 0.05, **P < 0.01, ***P < 0.001. Supplementary Fig. 5: (A-B) Western blotting analysis of GPX4 protein expression in sh-MRPL36-treated LN229 (A) and U251MG (B) cells after knockdown of HERC2, TRIM47, TRIM33, or UBR4. (C-D) Co-IP assay for GPX4 ubiquitination levels in sh-MRPL36-treated LN229 (C) and U251MG (D) cells after si-TRIM33 knockdown. (E) Co-IP assay for GPX4 ubiquitination levels in sh-MRPL36-treated U251MG cells after treatment with PA, 2-BP, or si-TRIM33 as indicated. (F-G) Co-IP assay for GPX4 ubiquitination levels in control LN229 (F) and U251MG (G) cells after treatment with 2-BP or si-TRIM33 as indicated. *P < 0.05, **P < 0.01, ***P < 0.001. Supplementary Fig. 6: (A-B) Representative images of EdU fluorescence staining (A) and quantitative analysis (B) for the effect of MRPL36 knockdown alone or combined with TRIM33 knockdown on cell proliferation in LN229 and U251MG cells. (C-D) Representative images of wound healing assay (C) and quantitative analysis (D) for the effect of MRPL36 knockdown alone or combined with TRIM33 knockdown on cell migration in LN229 and U251MG cells. (E-F) Representative images of Transwell migration/invasion assay (E) and quantitative analysis (F) for the effect of MRPL36 knockdown alone or combined with TRIM33 knockdown on cell migration and invasion in LN229 and U251MG cells. *P < 0.05, **P < 0.01, ***P < 0.001


## Data Availability

The data analyzed in this research can be found in the CGGA (http://www.cgga.org.cn/) and GEO (https://www.ncbi.nlm.nih.gov/gds) websites.

## Abbreviations

GBM: Glioblastoma Multiforme
MRPL36: Mitochondrial Ribosomal Protein L36
GPX4: Glutathione Peroxidase 4
TRIM33: Tripartite Motif-Containing 33
PCTs: Paracancerous Tissues
NBT: Normal Brain Tissue
IHC: Immunohistochemistry
PA: Palmitic Acid
ZDHHC12: Zinc Finger DHHC-type palmitoyltransferase 12
2-BP: 2-Bromopalmitate
CHX: Cycloheximide
MDA: Malondialdehyde
GSH: Glutathione
GSSG: Glutathione disulfide
Fer-1: Ferrostatin-1
PBS: Phosphate Buffered Saline
ROS: in Reactive Oxygen Species
TEM: Transmission Electron Microscopy
K48: Lysine 48
Ub: Ubiquitin
ACSL4: Acyl-CoA Synthetase Long-Chain Family Member 4
4-HNE: 4-Hydroxynonenal
