# Supplementary material for: Dual regulation of metabolic reprogramming and protein competitive modifications: the core role and mechanism of MRPL36 in glioblastoma malignant progression
Source: J Exp Clin Cancer Res. 2026 Jun 18;45:189. doi: 10.1186/s13046-026-03764-w (PMC13523217; doi:10.1186/s13046-026-03764-w)
Supplement: Supplementary file 1 [file 13046_2026_3764_MOESM1_ESM.docx]

**Supplementary Figure 1:** (A) Construction of a nude mouse model of intracranial xenograft tumor. (B-C) Intracranial tumor fluorescence intensity and volume were observed and evaluated by *in vivo* imaging and HE staining. (D) A line chart was used to analyze the difference in total fluorescence intensity in nude mice between the control and sh-MRPL36 groups. (E) A line chart compared body weight changes in the two groups of nude mice at different time points. (F) Survival analysis was performed on tumor-bearing mice in both groups. (G-H) IHC was used to detect the differential expression of Ki67 in tumor tissues of the two groups. **P < 0.05, **P < 0.01, ***P < 0.001.*

**Supplementary Figure 2:** (A) Volcano plot of differentially expressed genes between control and sh-MRPL36 groups. (B-C) GO/KEGG enrichment analyses showing significant changes in ferroptosis-related pathways. (D) Non-targeted metabolomics revealing differential expression of palmitoyl-CoA. (E-F) Representative ROS fluorescence images (E) and quantitative analysis (F) of four cell groups. (G-H) Representative Fe^2+^ fluorescence images (G) and quantitative analysis (H) of four cell groups. (I-K) Western blotting (I) and quantitative statistics (J, K) of GPX4 and ACSL4 expression in four cell groups. **P < 0.05, **P < 0.01, ***P < 0.001.*

**Supplementary Figure 3:** (A) Transmission electron microscopy images of mitochondrial ultrastructure in U251MG cells. (B-E) Colony formation assay in control and MRPL36-knockdown LN229 and U251MG cells treated with PBS or Fer-1, with representative images (B, C) and quantitative bar graphs (D, E). (F-I) EdU proliferation assay in the aforementioned four groups of LN229 and U251MG cells, with representative fluorescence images (F, G) and statistical graphs (H, I). (J-M) Wound healing assay to evaluate the migratory ability of the aforementioned four groups of LN229 and U251MG cells, with representative images (J, K) and statistical graphs (L, M). (N-Q) Transwell assay of migratory and invasive abilities in the aforementioned four groups of LN229 and U251MG cells, with representative images (N, O) and quantitative bar graphs (P, Q). **P < 0.05, **P < 0.01, ***P < 0.001.*

**Supplementary Figure 4:** (A-B) CHX chase assay (2.5 µg/mL CHX) for GPX4 protein stability in control and MRPL36-knockdown U251MG cells at 0, 2, 4, and 8 hours: representative Western blotting images (A) and quantitative analysis (B). (C) ABE assay for GPX4 palmitoylation levels in control and MRPL36-knockdown U251MG cells with or without HAM treatment. (D-E) Relative cell viability of control and MRPL36-knockdown LN229 (D) and U251MG (E) cells treated with palmitic acid (0, 25, 50, 100 µM) for 6 hours. (F) ABE assay for GPX4 palmitoylation in sh-MRPL36-treated U251MG cells after treatment with PA, 2-BP, and HAM as indicated. (G-K) Survival prognosis analysis of ZDHHC8 (G), ZDHHC12 (H), ZDHHC18 (I), ZDHHC21 (J), and ZDHHC24 (K) was conducted based on the SangerBox database. (L) ABE assay for GPX4 palmitoylation in sh-MRPL36-treated U251MG cells treated with PA, HAM, 2-BP, or si-ZDHHC (8, 12, 18, 21, 24) as indicated. (M) ABE assay for Flag-GPX4 palmitoylation in control and MRPL36-knockdown U251MG cells expressing wild-type (WT), C66A, or C75A Flag-GPX4 after PA and HAM treatment. (N-O) CHX chase assay (2.5 µg /mL CHX) for GPX4 protein stability in sh-MRPL36-treated U251MG cells expressing wild-type (WT) or C66A Flag-GPX4 at 0, 2, 4, and 8 hours: representative Western blot images (N) and quantitative analysis (O). (P) Co-IP assay for GPX4 ubiquitination levels in control and MRPL36-knockdown U251MG cells. (Q) Co-IP assay for GPX4 ubiquitination levels in sh-MRPL36-treated U251MG cells after treatment with PA, 2-BP, or si-ZDHHC12. (R) Co-IP assay for GPX4 ubiquitination in sh-MRPL36-treated U251MG cells expressing wild-type (WT) or C66A Flag-GPX4 after treatment with PA, 2-BP, or si-ZDHHC12 as indicated. **P < 0.05, **P < 0.01, ***P < 0.001.*

**Supplementary Figure 5:** (A-B) Western blotting analysis of GPX4 protein expression in sh-MRPL36-treated LN229 (A) and U251MG (B) cells after knockdown of HERC2, TRIM47, TRIM33, or UBR4. (C-D) Co-IP assay for GPX4 ubiquitination levels in sh-MRPL36-treated LN229 (C) and U251MG (D) cells after si-TRIM33 knockdown. (E) Co-IP assay for GPX4 ubiquitination levels in sh-MRPL36-treated U251MG cells after treatment with PA, 2-BP, or si-TRIM33 as indicated. (F-G) Co-IP assay for GPX4 ubiquitination levels in control LN229 (F) and U251MG (G) cells after treatment with 2-BP or si-TRIM33 as indicated. **P < 0.05, **P < 0.01, ***P < 0.001.*

**Supplementary Figure 6:** (A-B) Representative images of EdU fluorescence staining (A) and quantitative analysis (B) for the effect of MRPL36 knockdown alone or combined with TRIM33 knockdown on cell proliferation in LN229 and U251MG cells. (C-D) Representative images of wound healing assay (C) and quantitative analysis (D) for the effect of MRPL36 knockdown alone or combined with TRIM33 knockdown on cell migration in LN229 and U251MG cells. (E-F) Representative images of Transwell migration/invasion assay (E) and quantitative analysis (F) for the effect of MRPL36 knockdown alone or combined with TRIM33 knockdown on cell migration and invasion in LN229 and U251MG cells. **P < 0.05, **P < 0.01, ***P < 0.001.*
